# *Lactobacillus plantarum*-derived postbiotics prevent *Salmonella*-induced neurological dysfunctions by modulating gut–brain axis in mice

**DOI:** 10.3389/fnut.2022.946096

**Published:** 2022-07-28

**Authors:** Yanping Wu, Yan Wang, Aixin Hu, Xin Shu, Wenxia Huang, Jinsong Liu, Baikui Wang, Ruiqiang Zhang, Min Yue, Caimei Yang

**Affiliations:** ^1^College of Animal Science and Technology, College of Veterinary Medicine, Zhejiang Agricultural and Forestry University, Hangzhou, China; ^2^Zhejiang Vegamax Biotechnology Co., Ltd., Huzhou, China; ^3^College of Animal Sciences, Zhejiang University, Hangzhou, China

**Keywords:** *Lactobacillus plantarum*, postbiotics, *Salmonella*, neurological dysfunctions, gut–brain axis

## Abstract

Postbiotics are the inactive bacteria and/or metabolites of beneficial microbes which have been recently found to be as effective as their live probiotic. This study aimed to evaluate the benefits of *Lactobacillus plantarum* (LP)-derived postbiotics on ameliorating *Salmonella*-induced neurological dysfunctions. Mice were pretreated with LP postbiotics (heat-killed bacteria or the metabolites) or active bacteria, and then challenged with *Salmonella enterica* Typhimurium (ST). Results showed that LP postbiotics, particularly the metabolites, effectively prevented ST infection in mice, as evidenced by the inhibited weight loss, bacterial translocation, and tissue damages. The LP postbiotics markedly suppressed brain injuries and neuroinflammation (the decreased interleukin (IL)-1β and IL-6, and the increased IL-4 and IL-10). Behavior tests indicated that LP postbiotics, especially the metabolites, protected mice from ST-induced anxiety and depressive-like behaviors and cognitive impairment. A significant modulation of neuroactive molecules (5-hydroxytryptamine, gamma-aminobutyric acid, brain-derived neurotrophic factor, dopamine, acetylcholine, and neuropeptide Y) was also found by LP postbiotic pretreatment. Microbiome analysis revealed that LP postbiotics optimized the cecal microbial composition by increasing *Helicobacter, Lactobacillus* and *Dubosiella*, and decreasing *Mucispirillum*, norank_f_Oscillospiraceae, and Eubacterium_siraeum_group. Moreover, LP postbiotics inhibited the reduction of short-chain fatty acids caused by ST infection. Pearson's correlation assays further confirmed the strong relationship of LP postbiotics-mediated benefits and gut microbiota. This study highlights the effectiveness of postbiotics and provide a promising strategy for preventing infection-induced brain disorders by targeting gut–brain axis.

## Introduction

Bacterial infection, as one of the main threats to humans and animals, causes mounting public health concerns and enormous medical and financial burdens annually (1). Most invasive bacteria can lead to a systemic infection and serious complications. In the recent years, accumulating evidence has highlighted the ever-enhancing complications associated with neurological manifestations (2). Bacterial infection activates neural and central nervous system (CNS) signaling systems, and consequently causes brain dysfunction, behavior disorders, and mental diseases (3). For example, infection with *Campylobacter jejuni, Citrobacter rodentium*, and *Staphylococcus aureus* were found to induce anxiety-like behavior, memory impairment, brain permeability, and inflammation (4–6). This situation occurs through various mechanisms, including altering hormonal and neurotransmitter communication, increasing neuroinflammation, and affecting the structure and functions of neurons and brain (7, 8). Antibiotic is the main therapy approach for bacterial infection. However, the overuse of antibiotics has led to the increasing emergence of multiantibiotic-resistant bacteria. Furthermore, the neurological complications cannot be completely treated by antibiotics and the cure rate remains alarmingly poor (9). The situation, more so in infants, presents with a poor prognosis, and relatively high mortality rate (10). Also, early exposure to antibiotic drugs elevates risks for a spectrum of mental disorders (11). Apparently, antibiotic treatment is inefficacious and have many side effects, thus it is urgent to evaluate other strategies as a potential treatment option.

“Gut–brain axis” refers to the bidirectional communication that occurs between CNS and gastrointestinal tract (12). It comprises the CNS, neuroimmune, and neuroendocrine systems, autonomic nervous system and gut microbiota (13). Growing evidence reported that modulation of gut microbiota is suggested to be an effective strategy for maintaining neurodevelopment and brain functions, and treating mental disorders (12). Probiotics are well-known for their ability to optimize gut microbiota to exert benefits. The recent studies revealed that probiotics can reverse neurological dysfunction, and therefore, a new term “psychobiotics” occurs to highlight probiotics that, upon ingestion in adequate amounts, yield positive influence on mental health (14). For instance, administered with *Lactobacillus rhamnosus* alleviated signs of anxiety and depression in mice (15); *Lactobacillus plantarum* rescued cognitive dysfunctions, and alleviated the reduced levels of neurotransmitters in mice during chronic stress (16); *Lactobacillus casei* improved depression-like behavior in mild stress-induced rats (17). It was reported that probiotics drive CNS functions mainly depending on altering the composition and metabolism of gut microbiota (18). The microbial components and metabolites cross the blood–brain barrier to modulate the brain functions and neurotransmitter levels (19). Among them, tryptophan metabolites, 5-hydroxytryptamine (5-HT), gamma-aminobutyric acid (GABA), brain-derived neurotrophic factor (BDNF), branched-chain amino acid, short-chain fatty acids (SCFAs), and bile acids are the primary host/microbes-derived molecules (20).

Nevertheless, as most probiotics are restricted in storage and low survival in hostile environment, researchers are paying increasing attention to the functions of their components (inactive bacteria and metabolites) (21). These components are now called “postbiotics,” defined as “preparation of inanimate microorganisms and/or their components that confers a health benefit on the host” (22). Accumulating studies have revealed that postbiotics are equivalent or even superior to the live probiotics (23–25). The ability of postbiotics in inhibiting bacterial infection has been proved by the recent studies (26). However, by far, few reports have explored their effects on pathogen-induced neurological dysfunctions, and the underlying mechanism is obscure. *Salmonella* is the main food-derived pathogen that claims thousands of lives worldwide. *Salmonella* can invade the brain tissue, and reports have indicated neurological manifestations in patients suffering during the course of *Salmonella* infection and afterward (2). A recent study showed that probiotic intervention prevented serious manifestations during *Salmonella* and acted as a potential psychobiotic (27). We previously found that the metabolites of a probiotic strain *L. plantarum* HJZW08 showed strong antibacterial activity on *Salmonella* Typhimurium, and the effect was better than *L. rhamnosus* and *L. paracasei*, indicating that LP exhibits a superior anti-*Salmonella* capacity. On this base, this study aimed to explore the efficacy of LP postbiotics on ameliorating *Salmonella*-induced neurological dysfunctions in comparison to its active bacteria, and tried to clarify the underlying mechanisms from the perspective of modulating gut–brain axis.

## Materials and methods

### Bacteria preparation

The probiotic *L. Plantarum* HJZW08 (LP), obtained from Zhejiang Vegamax Biotechnology Co., Ltd, was anaerobically and statically cultured in MRS broth at 37°C for 24 h. The LP active bacteria (LPB_active_) were prepared as follows: The 24-h-cultured LP was centrifuged at 8,000*g* at 4°C for 10 min, washed 3 times with PBS, resuspended in PBS, detected at OD_600_ using a microplate reader (BioTek Synergy H1, BioTek Instruments, Winooski, VT, USA), and then the concentration was adjusted to 1 × 10^9^ cfu/ml according to the OD_600_-concentration linear relation; thereafter, the bacteria were centrifuged and resuspended in the same volume of fresh MRS broth. For the inactive LP (LPB_inactive_) preparation, the diluted LP at the concentration of 1 × 10^9^ cfu/ml was heat-killed at 100°C for 30 min, centrifuged and finally resuspended in the fresh MRS broth. The metabolites of LP (LP cell-free culture supernatants, LPC) were obtained by centrifugation of the 24-h-cultured LP bacterial suspensions at 8,000*g* at 4°C for 10 min, collection of the supernatants, and then filtration by 0.22-μm membrane filters (Millex–GP, Millipore, Bedford, MA, USA); thereafter, the supernatants were diluted as the same fold as the LP bacteria (the fold that LP adjusted to 1 × 10^9^ cfu/ml) with MRS broth. *Salmonella enterica* Typhimurium SL1344 (ST) was cultured overnight in LB broth at 37°C, washed with PBS and resuspended at 3 × 10^9^ cfu/ml.

### Animal experimental design

**Figure 1:**
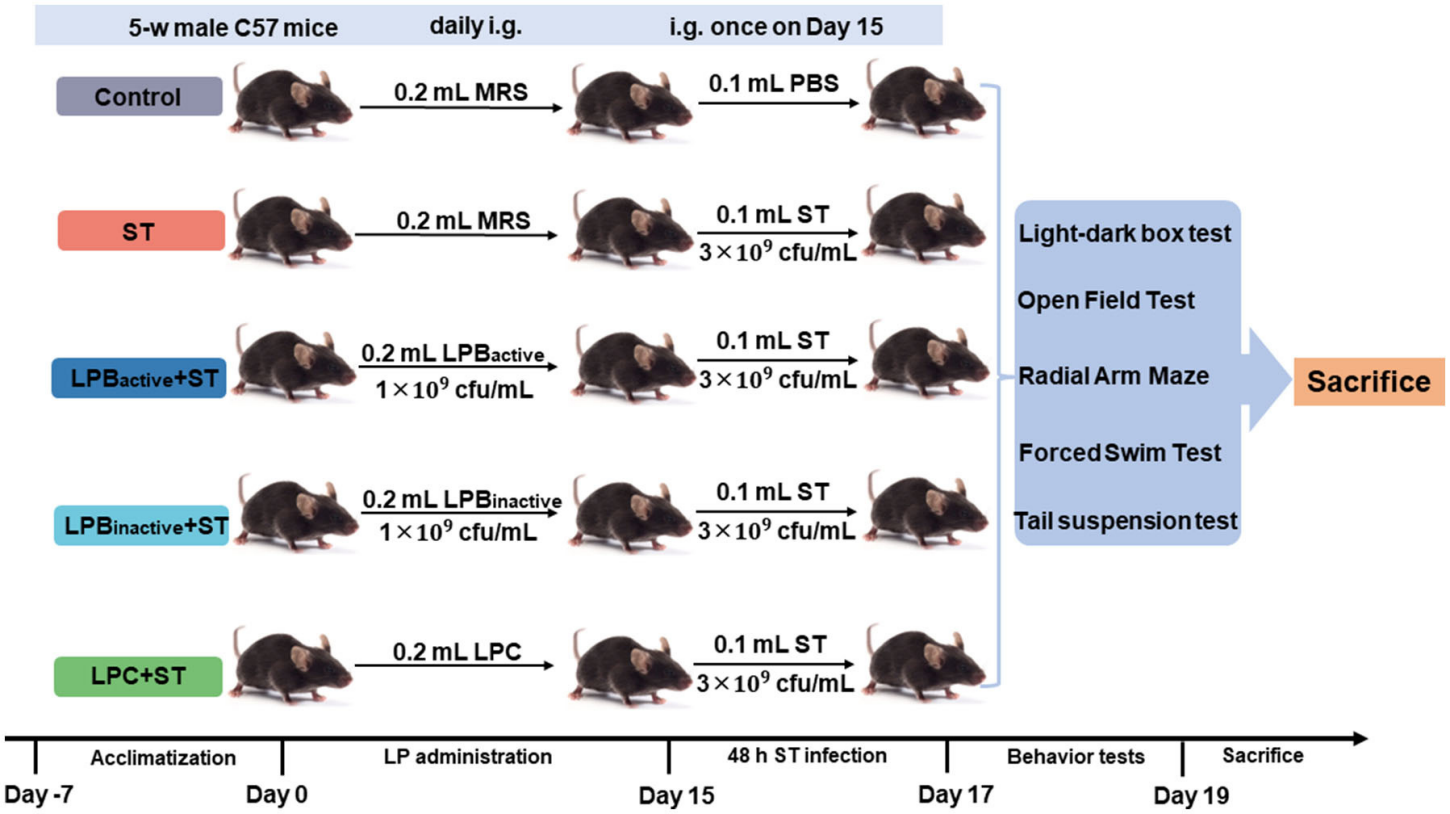
Schematic diagram displaying the experimental design. The experiment was conducted in three sequential stages. Mice were intragastrically gavaged (i.g.) daily with 0.2 ml of LP postbiotics for 15 days, and then i.g. with 0.1 ml of 3 × 10^9^ cfu/ml ST. Forty-eight hours post infection, mice were carried out to do behavior tests for 2 days, and were finally sacrificed the next day.

The animal study was approved by the Animal Care and Use Committee of Zhejiang Agricultural and Forestry University. Eighty 5-week-old male C57/BL6 mice were obtained from SLAC Laboratory Animal Co., Ltd (Shanghai, China). Mice were fed with a basal diet and housed in an airconditioned room on a 12-h light/dark cycle. Figure 1 displays the schematic diagram of the experimental design. After 7-day acclimatization, all 80 mice were randomly divided into 5 groups (Control, ST, LPB_active_ + ST, LPB_inactive_ + ST, and LPC + ST, *N* = 16 in each group), and intragastrically gavaged daily with 0.2 ml of MRS broth, LPB_active_ (1 × 10^9^ cfu/ml), LPB_inactive_ (1 × 10^9^ cfu/ml) and LPC, and the dose was selected according to the previous studies (28, 29). The body weight and feed intake were calculated every 3 days. After 15 days of pretreatments, 4 groups (ST, LPB_active_ + ST, LPB_inactive_ + ST, and LPC+ST) were intragastrically gavaged with 0.1 ml of 3 × 10^9^ cfu/ml ST (the dose was determined by a pre-test), and the Control group was gavaged with the same volume of PBS. Also, 48-h post infection, eight mice from each group were carried out to do behavior tests for 2 days, and thereafter, all mice were sacrificed the next day.

### Behavior tests

#### Light–dark box test

The apparatus consists of two chambers with lid separated by a connection door (100 × 50 mm). The light chamber (600 × 200 × 200 mm) was entirely white and illuminated with 400 lux, while the other one (200 × 200 × 200 mm) was entirely darkened. Mice were put in the box half an hour to adapt the environment. Then, the mouse was individually put into the light chamber facing the door, and allowed to freely explore the whole apparatus for 10 min. All trials were video-recorded from a digital camera mounted above the box and connected to a computer. The transition between two chambers, time spent in the light chamber, and the number of transfers were calculated. All of the sessions were recorded by a camera that was connected with a monitor, and data were analyzed by the ANY-maze software, version 6.3 (Stoelting, Wood Dale, IL, USA).

#### Open field test

The OFT consists of a square box (600 × 600 × 200 mm). Mice were put in the box half an hour to adapt the environment. Then, each mouse was placed in the center of the box, and its behavior was video-recorded for 10 min. The total distance traveled, the number of entries to the center, the time spent in the center, the average speed and the number of line crossings were recorded using a digital video tracking system (Viewpoint, Lissieu, France). Data were analyzed by the ANY-maze software, version 6.3 (Stoelting, Wood Dale, IL, USA).

#### Radial arm maze

The RAM consisted of eight arms extending radially from a central area. Four of which had 14-cm high walls (closed arms) and the other four had no walls (open arms) (Med Associates Inc., St. Albans, VT, USA). Before ST infection, mice were trained to visit the radial arms daily by feed reward method for a week. For the final test, the mouse was placed in a closed arm, and the movement around the maze was video-recorded from a digital camera mounted above the maze and connected to a computer for 5 min. The time of total visit task, open arm entries, mean of working, and reference memory errors were evaluated by a blinded observer to assess the behavior. Data were analyzed by the ANY-maze software, version 6.3 (Stoelting, Wood Dale, IL, USA).

#### Forced swim test and tail suspension test

Mice were trained 24 h before the tests. For FST, the mouse was forced to swim for 6 min in a 15-cm diameter apparatus filled with water (temperature: 23 ± 2°C) up to a depth of 15 cm. After 2-min acclimatization, the duration for which the mice remained immobile in water during the remaining 4 min was recorded. For TST, the mouse was hanged by the tail and its body hanged down in the air. The head was about 30 cm away from the table. After 2-min adaptation, the immobility time was recorded within 4 min. Mice were regarded immobile only when they hung passively and completely motionless.

### Sample collection

Blood was obtained from mouse heart and then blood serum was separated by centrifugation at 4,000*g* for 10 min at 4°C. The whole brain tissue was separated, and one quarter was immersed in 4% paraformaldehyde for morphologic analysis while the remaining was for ELISA detection. The colon and spleen were dissected from the body and photographed using camera, and the length or weight was calculated. A small segment of the tissues was excised for histological analysis and bacteria counting. Cecal contents were collected for SCFA measurement and 16S rRNA sequencing. The samples were stored at −80°C until further detection.

### Measurement of *Salmonella* colonization

The colonization of *Salmonella* in the colon, spleen, and brain were detected using agar plate method. Briefly, tissue samples were homogenized in PBS containing 0.1% Triton X-100 and serially diluted by 10-fold. The diluted tissue homogenates were coated on SS agar plates and cultured for 24 h at 37°C. Bacteria colonies were counted and the number of bacteria colonization were calculated by per gram tissue.

### Analysis of tissue morphology

After fixed by 4% paraformaldehyde, colon, spleen, and brain samples were dehydrated in ethanol, infiltrated with xylene and embedded in paraffin blocks according to a standard procedure. The paraffin sections were sliced and subjected with hematoxylin & eosin (H&E). Tissue structure was observed and images were taken using Nikon optical microscope system (Tokyo, Japan).

### Measurement of inflammatory cytokines and neuroactive molecules

The levels of inflammatory cytokines interleukin (IL)-1β, IL-4, IL-6, and IL-1; and tumor necrosis factor (TNF-α), and neuroactive molecules including 5-HT, GABA, BDNF, dopamine (DA), acetylcholine (ACH) and neuropeptide Y (NPY) were detected by ELISA kits following the manufacturer's instructions (Angle Gene, Nanjing, China).

### Detection of SCFA concentration

The concentration of SCFAs (acetatic acid, propionic acid, butyric acid, isobutyric acid, valeratic acid, and isovaleric acid) was detected using the headspace sampler gas chromatography based on our previous study (30). Briefly, 0.3-g cecal contents from each sample were diluted with 1.2 ml sterile water and centrifuged at 12,000*g* for 10 min at 4°C. The supernatant was mixed with metaphosphoric acid (m/v, 1: 4), and then injected into an Agilent Technologies GC7890 Network System with a flame ionization detector (Agilent Technologies, Wilmington, DE, USA) for detection.

### Analysis of 16S rRNA-based microbiome

The genomic DNA of cecal samples was extracted using E.Z.N.A.^®^ soil DNA Kit (Omega Bio-tek, Norcross, GA, USA) according to the manufacturer's protocol. The quality and quantity of DNA were determined before PCR amplification. The V3–V4 variable regions of the 16S rRNA genes were amplified with normal primers 338F (5'-ACTCCT ACGGGAGGCAGCA-3') and 806R (5'-GGACTACHVGGGTWTCTA AT-3'). The PCR products were purified and then pooled in equimolar amounts and paired-end sequenced on the Illumina MiSeq platform (Illumina, San Diego, CA, USA), as per the instructions of Majorbio Bio-Pharm Technology Co. Ltd. (Shanghai, China).

Raw fastq files were filtered by Trimmomatic and merged by FLASH1.2.11. Operational taxonomic units (OTUs) were clustered with 97% similarity using UPARSE software (version 7.1, http://drive5.com/uparse/). The taxonomy of 16S rRNA sequences was analyzed using RDP Classifier algorithm (https://sourceforge.net/projects/rdp-classifier/) against the database with the confidence threshold of 70%. α diversity was performed using Mothur1.30.2 software (https://www.mothur.org/wiki/Download_mothur). The β -diversity, displayed as principal co-ordinates analysis (PCoA), was analyzed based on the weighted UniFrac distance and ANOSIM using QIIME1.9.1. The composition of microbiota was performed based on tax_summary and R package (version 3.3.1). The differences of microbiota composition at genus level between groups was performed by STAMP software (version 2.1.3) and analyzed by Student's *t*-test.

The network was performed to study the co-occurrence patterns of bacterial taxa. The genera with relative abundance above 0.05% were screened. The Spearman's correlation between two genera was identified statistically significant (28). Each node indicates one genus, and each edge represents a notable correlation among the nodes. The parameters (nodes, edges, average degree, network diameter, graph density, modularity, average path length, positive correlation, and negative correlation) were determined using igraph packages in R environment to clarify the topology of the networks (31). The networks were visualized in the interactive platform Gephi 0.9.2.

### Statistical analysis

Data were presented as Mean ± SEM, and analyzed by one-way ANOVA Tukey's tests using IBM SPSS (version 21.0, Chicago, IL, USA). Figures were drawn using GraphPad Prism 8.0. The correlation analyses were performed between significant microbes, SCFAs, weight loss, ST translocation in brain, inflammatory cytokines, and neuroactive molecules. Correlation coefficients were calculated using Mantel's and Pearson's correlation distance, and heatmaps were performed by R package to identify relationships between the variables. Moreover, *p* < 0.05 indicates the significant difference.

## Results

### The LP postbiotics effectively protect against ST infection in mice

**Figure 2:**
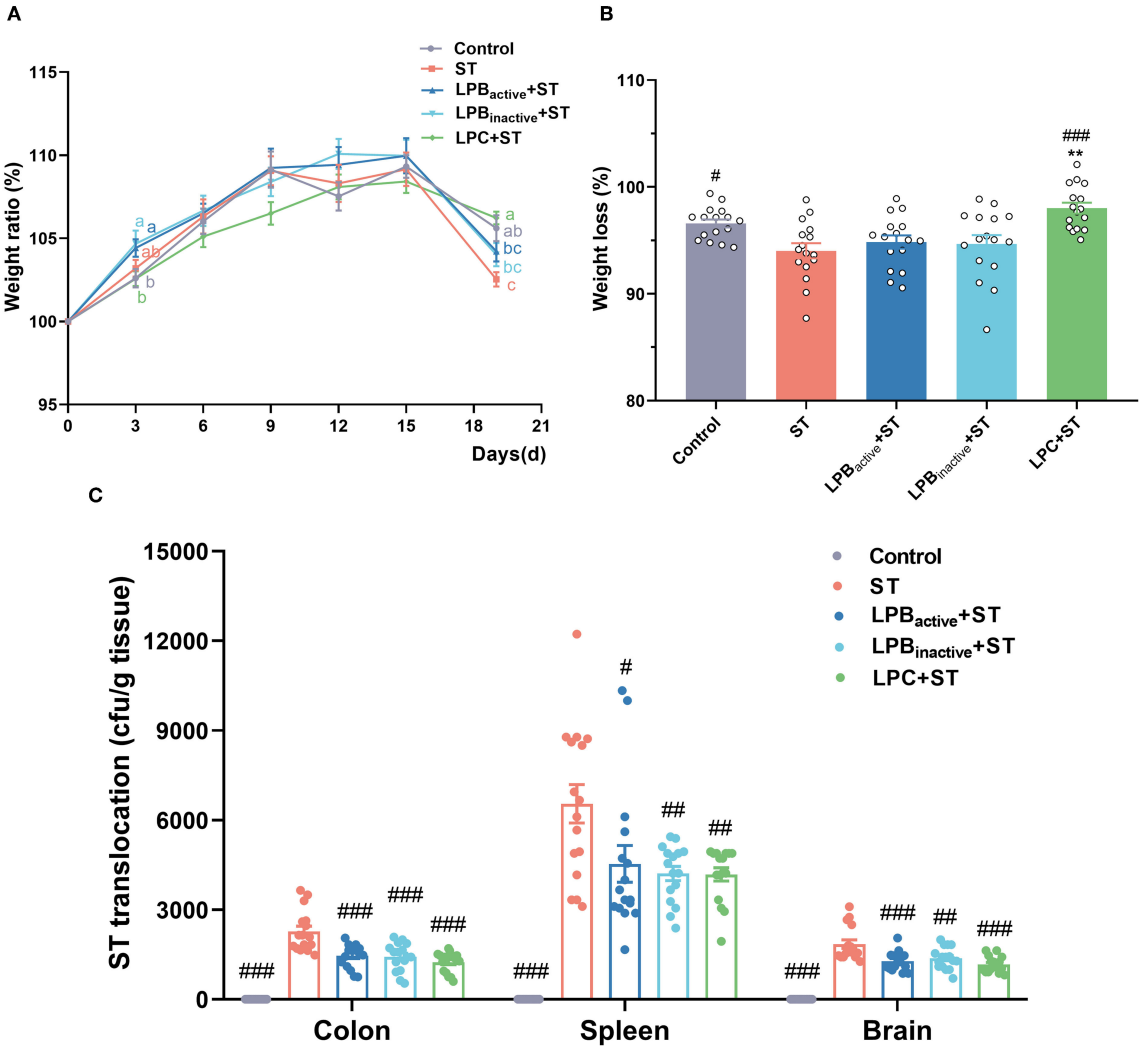
The LP postbiotics prevent ST infection in mice. **(A)** weight gain. The body weight of each mouse was measured every 3 days. Weight ratio was calculated as (body weight on day N)/(body weight on day 0) × 100% **(B)** Weight loss was calculated as (weight on day 19/weight on day 15) × 100%. **(C)** *Salmonella* translocation. S*almonella* translocation in the colon, spleen, and brain were detected using agar plate method, and the number of bacteria was calculated by per gram tissue. The data shown as mean ± SEM were analyzed using one-way ANOVA and Tukey's test (*N* = 16 in each group). Different letters (a, b, and c) represent significant (*p* < 0.05) and the color of letters represent the group with the same color. ^#^Indicates the significance compared to ST group: ^#^*p* < 0.05, ^##^*p* < 0.01, ^###^*p* < 0.001. *Indicates the significance compared to LPB_active_ + ST group: **p < 0.01.

As shown in Figure 2A, compared with the control, mice administrated LPB increased the weight ratio (weight on Day N/weight on Day 0 × 100%) along with the feeding day, particularly on Day 3 (*p* < 0.05), and LPB_inactive_ showed an almost equal effect with LPB_active_. Nevertheless, LPC exhibited a decreased trend but the differences were not significant (*p* > 0.05). Noticeably, a reduction of weight ratio was observed in Control, ST and LPB_active_ group. After ST challenge on Day 15, the weight ratio was dramatically decreased in ST group when compared to the Control group (*p* < 0.05) (Figures 2A,B). LPC pretreatment showed a strong capacity to reverse this trend (*p* < 0.001), while LPB_active_ and LPB_inactive_ exhibited no obvious changes (*p* > 0.05). Bacteria translocations were shown in Figure 2C. The results showed that LP postbiotics as well as its active bacteria dramatically decreased ST colonization in the colon (*p* < 0.001). In the spleen, a more significant inhibition of bacteria translocation was found by LPB_inactive_ and LPC pretreatments (*p* < 0.01) than LPB_active_ (*p* < 0.05). Bacteria translocation in the brain was also significantly suppressed by LP administration, especially LPB_active_ and LPC (*p* < 0.001). These findings suggested that LP postbiotics showed a similar effect in ameliorating ST translocation in mice.

### The LP postbiotics suppress tissue injury under ST challenge in mice

**Figure 3:**
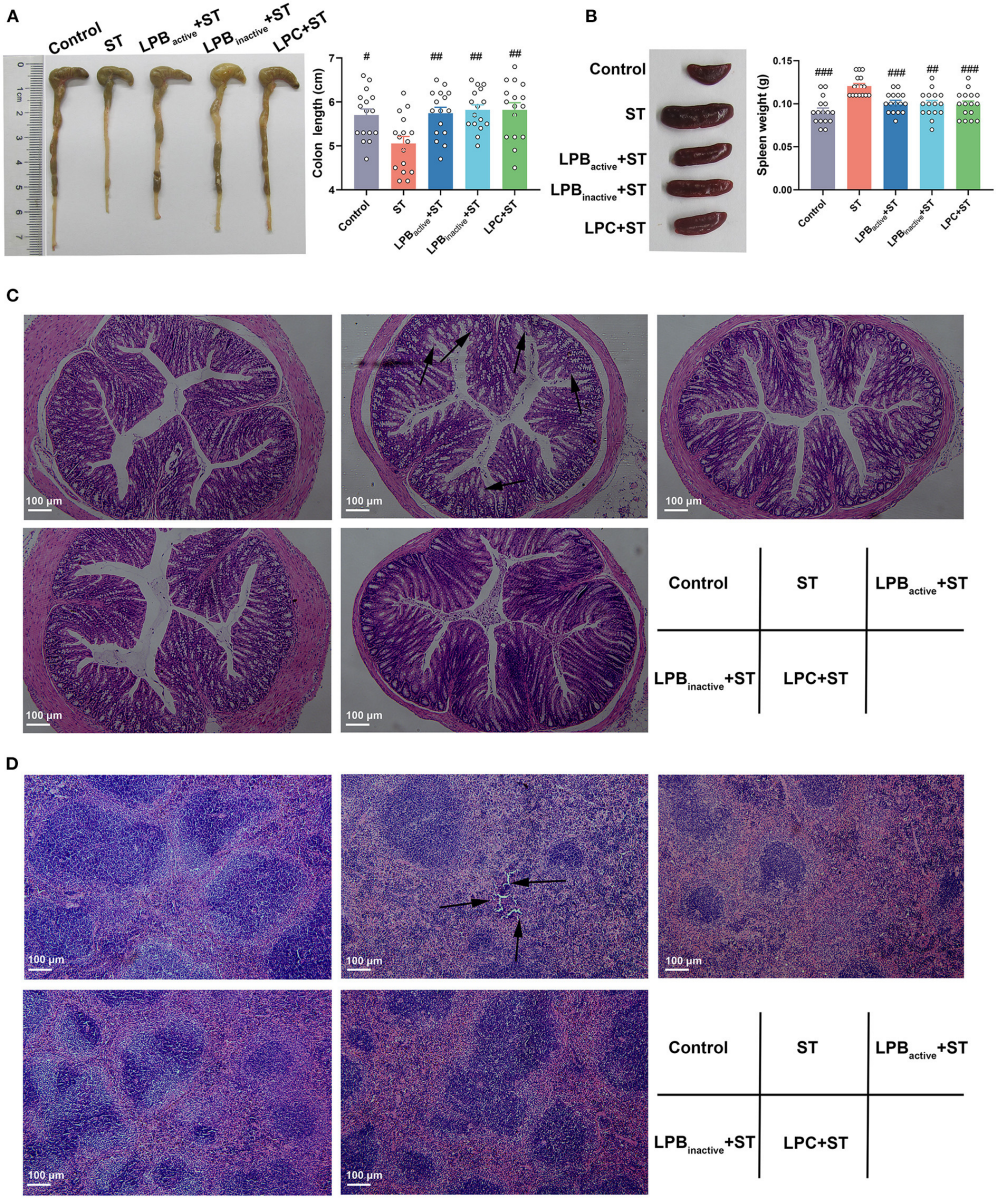
The LP postbiotics suppress tissue injury under ST challenge. **(A)** Pictures of colon and statistical analysis of colon length; **(B)** Pictures of spleen and statistical analysis of spleen weight. The data shown as mean ± SEM were analyzed by one-way ANOVA and Tukey's test (*N* = 16 in each group). ^#^Indicates the significance compared to ST group: ^#^*p* < 0.05, ^##^*p* < 0.01, ^###^*p* < 0.001. **(C,D)** Histomorphometric analysis of colon and spleen by H&E staining. 100 × magnification, scale bar: 100 μm. The arrows indicate the damaged spots.

The morphology of colon and spleen was shown in Figure 3. The colon structure became more vulnerable and the colon length was significantly decreased after ST challenge (*p* < 0.05); however, both LP postbiotics and its active probiotic administration significantly alleviated the injury (*p* < 0.01) (Figure 3A). The H&E staining also showed that the colon epithelial villi were damaged and incomplete in response to ST challenge, whereas LP pretreatments markedly reversed this trend (Figure 3C). Furthermore, the spleen in ST group was notably swelled, as evidenced by the significantly increased weight (*p* < 0.001), while LP postbiotics, particularly LPC dramatically decreased the degree of splenomegaly (*p* < 0.001) (Figure 3B). The histopathological changes including the broken structures were found in response to ST infection, but this alteration was inhibited by LP postbiotics, particularly the metabolites (Figure 3D).

### The LP postbiotics perform better than its active bacteria in preventing ST-induced brain injury and inflammation in mice

**Figure 4:**
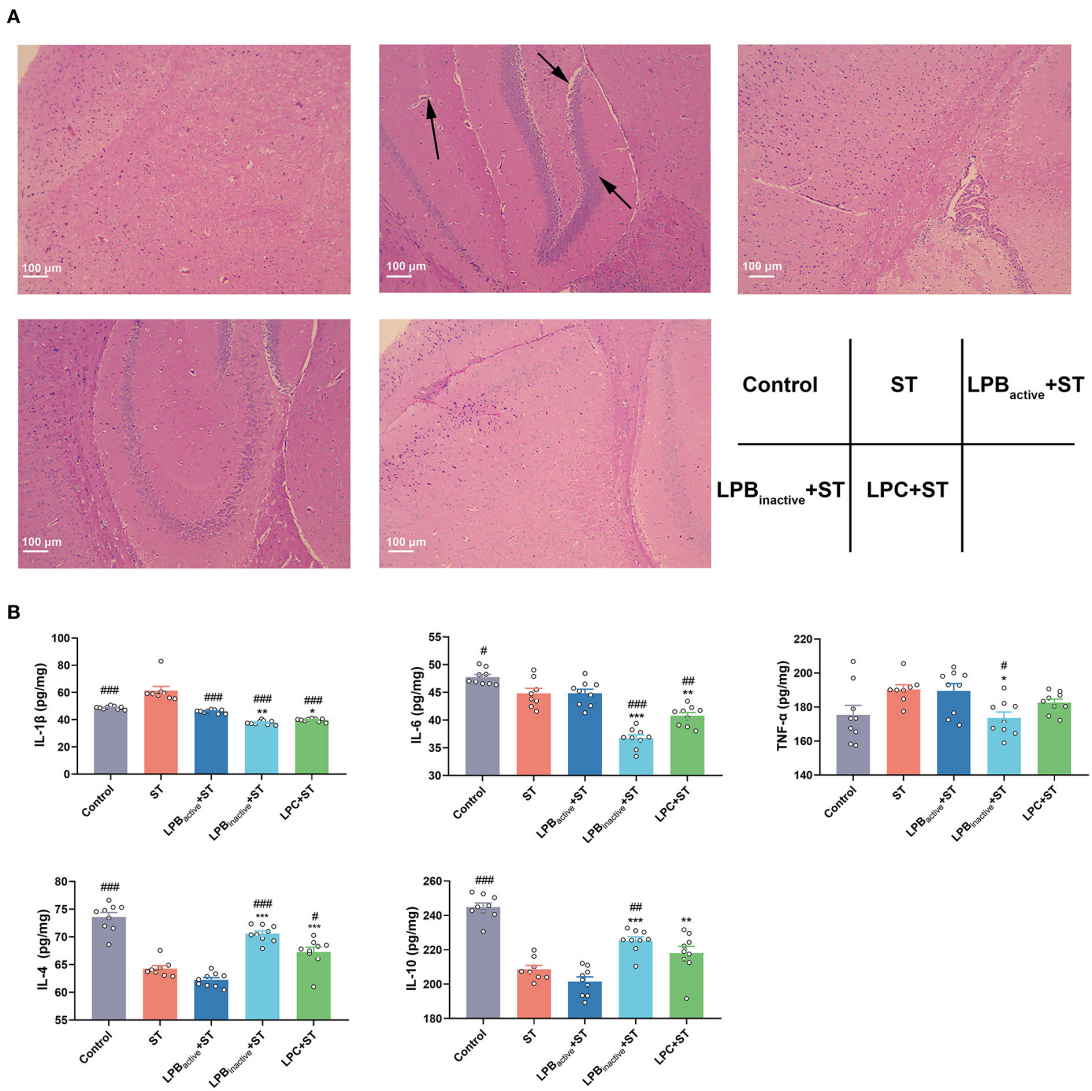
The LP postbiotics alleviate ST-induced brain injury and inflammation in mice **(A)** H&E staining of brain structure. 100 × magnification, scale bar: 100 μm. The arrows indicate the damaged spots. **(B)** Levels of inflammatory cytokines (IL-1β, IL-6, TNF-α, IL-4, and IL-10) in brain. The data shown as mean ± SEM were analyzed by one-way ANOVA and Tukey's test (*N* = 8 or 9 in groups). ^#^Indicates the significance compared to ST group: ^#^*p* < 0.05, ^##^*p* < 0.01, ^###^*p* < 0.001. *Indicates the significance compared to LPB_active_ + ST group: *p < 0.05, **p < 0.01, ***p < 0.001.

Figure 4A displays the H&E staining of brain structure. Compared to the Control group, ST infection obviously damaged the brain structure, and increased the inflammatory cell infiltration. However, the LP postbiotic pretreatments, particularly LPC, could suppress it. As shown in Figure 4B, mice exposed to ST exhibited a marked increase of the proinflammatory cytokine IL-1β expression (*p* < 0.001), whereas all the LP components significantly reversed the increased trend (*p* < 0.001). The LP postbiotics, particularly LPB_inactive_ remarkably reduced the levels of IL-6 and TNF-α caused by ST challenge (*p* < 0.001 and *p* < 0.05, respectively) (*p* > 0.05). A dramatical reduction of the anti-inflammatory cytokines (IL-4 and IL-10) was found in response to ST challenge (*p* < 0.001), while the decreased levels were inhibited by LPB_inactive_ and LPC pretreatments (IL-4, *p* < 0.001, *p* < 0.01, respectively; IL-10, *p* < 0.05, *p* > 0.05, respectively). Surprisingly, compared to the active bacteria, LP postbiotics showed a stronger capacity to modulate the inflammatory cytokines. To be specific, compared to LPB_active_, LPB_inactive_ significantly downregulated the concentrations of IL-1β, IL-6, and TNF-α (*p* < 0.01, *p* < 0.001, and *p* < 0.05, respectively), and upregulated IL-4 and IL-10 (*p* < 0.001); LPC markedly decreased the levels of IL-1β and IL-6 (*p* < 0.05 and *p* < 0.01), and increased IL-4 and IL-10 (*p* < 0.001 and *p* < 0.01). These results indicated that LP postbiotics exhibited better effects than the live probiotic in protecting against ST-induced brain injury and inflammation.

### The LP postbiotics, particularly the metabolites, inhibit ST-induced anxiety-like behaviors in mice

**Figure 5:**
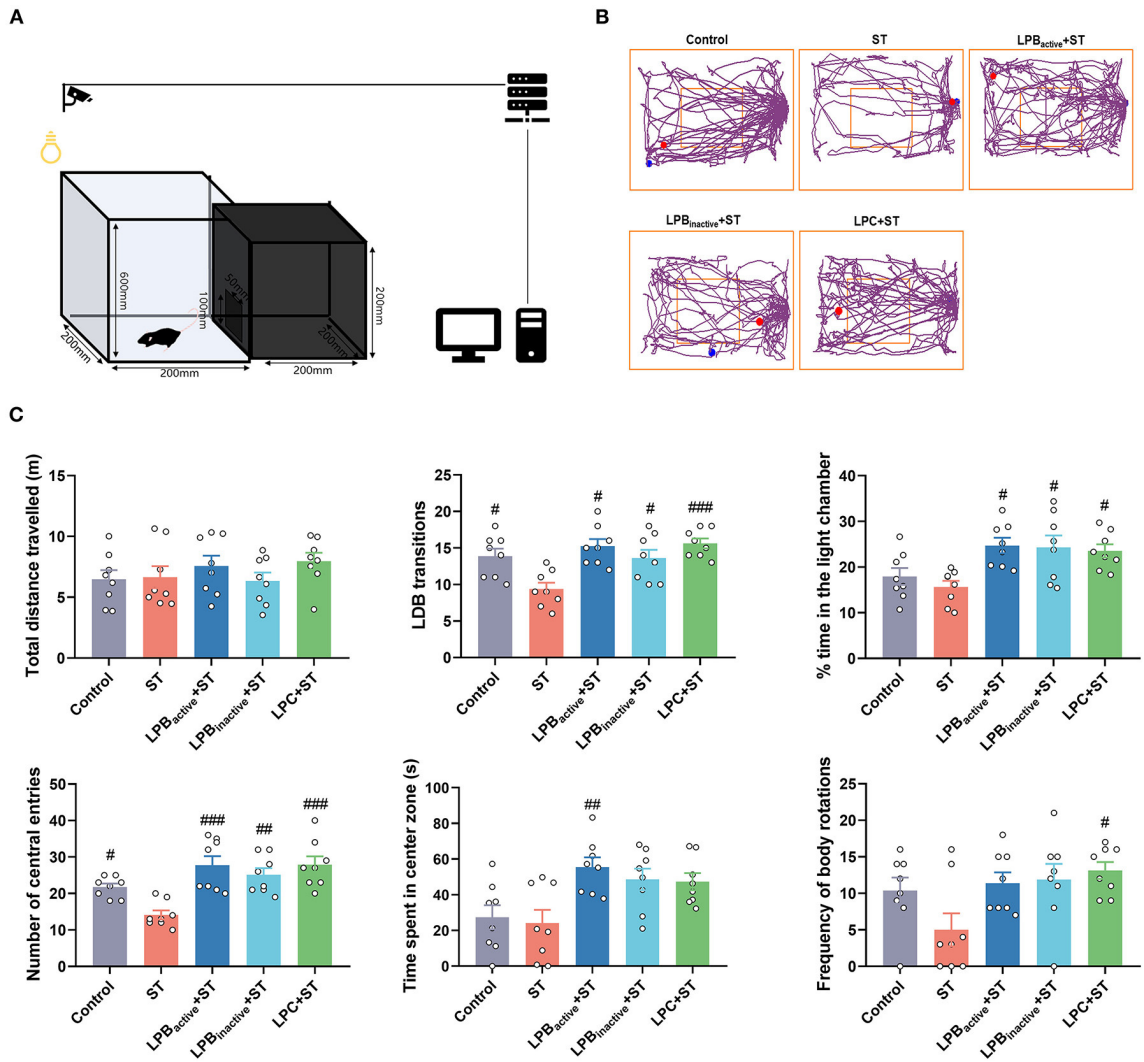
The light–dark box (LDB) test representing anxiety-like behaviors. **(A)** A cartoon of the apparatus for LDB test is shown. **(B)** Characteristic tracks of mice in the light box. The blue dot represents the start point and the red dot represents the end point of the mouse activity. **(C)** Main data of LDB test. Statistical analysis of the total distance, LDB transitions, time spent in the light chamber, and number of central entries, and time spent in center zone. The data shown as mean ± SEM were analyzed by one-way ANOVA and Tukey's test (*N* = 8 in each group). ^#^Indicates the significance compared to ST group: ^#^*p* < 0.05, ^##^*p* < 0.01, ^###^*p* < 0.001.

**Figure 6:**
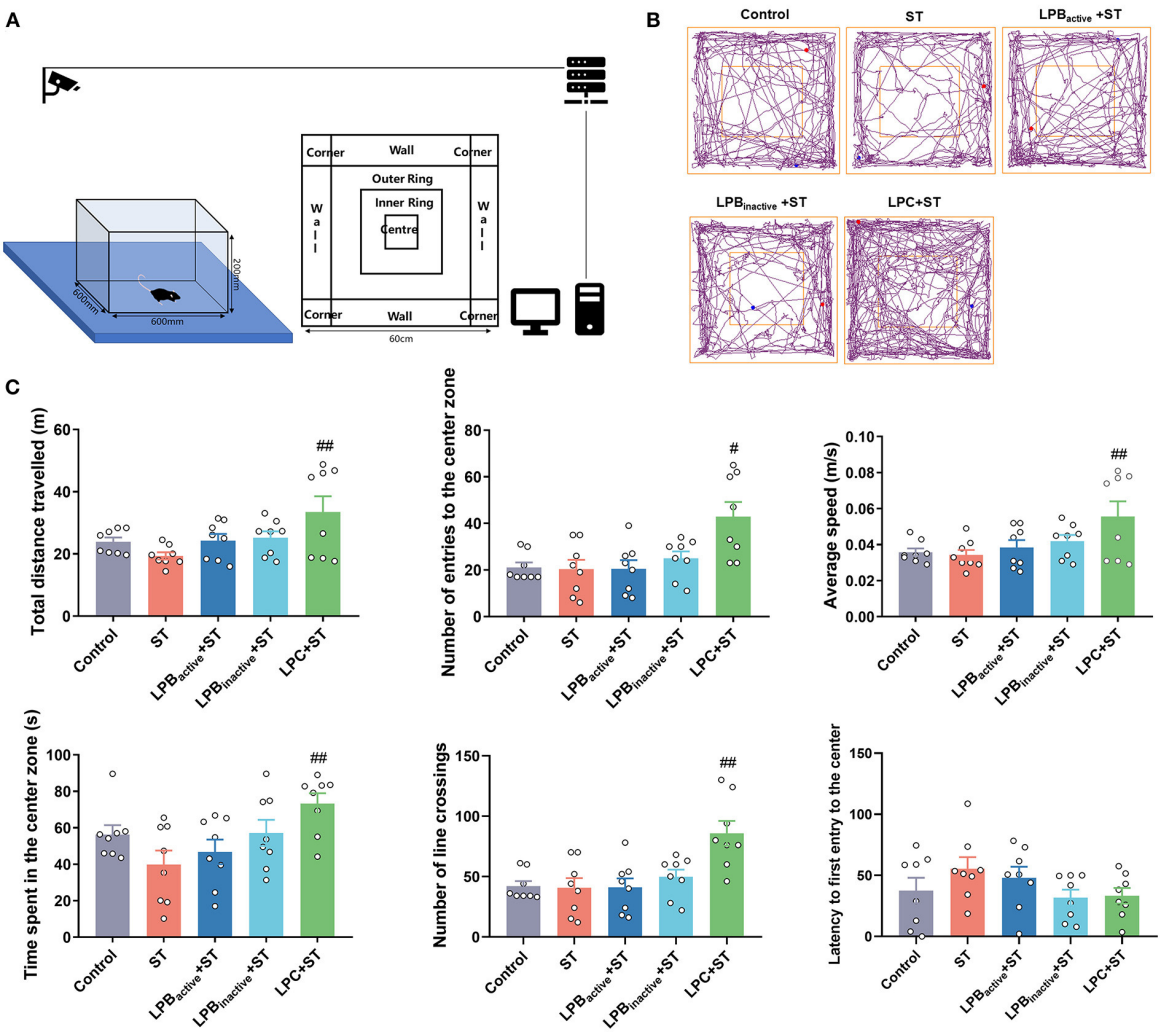
The open field test (OFT) representing anxiety-like behaviors. **(A)** A cartoon of the apparatus for OFT is shown. **(B)** Characteristic tracks of mice in the light box. The blue dot represents the start point and the red dot represents the end point of the mouse activity. **(C)** Main data of OFT. Statistical analysis of the total distance, number of entries to the center zone, average speed, time spent in the center zone, number of line crossing, and latency to first entry to the center. The data shown as mean ± SEM were analyzed by one-way ANOVA and Tukey's test (*N* = 8 in each group). ^#^Indicates the significance compared to ST group. ^#^*p* < 0.05, ^##^*p* < 0.01.

We then evaluated the anxiety-like behaviors by performing LDB and OFT tests. As shown in Figure 5, the LDB results revealed that no differences of total distance traveled was found among groups (*p* > 0.05). The ST challenge significantly decreased the number of transitions between light and dark chamber (*p* < 0.05), whereas LPB_active_, LPB_inactive_ and LPC pretreatments obviously increased it (*p* < 0.05, *p* < 0.05, and *p* < 0.001, respectively). The time spent in the light chamber was notably higher in all LP pretreated groups when compared to the ST group (*p* < 0.05). The ST infection dramatically decreased the number of central entries (*P* < 0.05), but this reduction was significantly reversed by LP postbiotics, particularly the metabolites (*p* < 0.01 and *p* < 0.001, respectively). The LPB_active_ pretreatment significantly increased the time spent in the center zone (*p* < 0.05), while LPC increased the frequency of body rotations under ST challenge (*p* < 0.05). In the OFT (Figure 6), only LPC pretreatment showed obvious changes. To be specific, compared to the ST group, LPC markedly upregulated the total distance traveled (*p* < 0.01), the number of entries to the center zone (*p* < 0.05), the average speed (*p* < 0.01), time spent in the center zone (*p* < 0.01) and number of line crossings (*P* < 0.01) during ST infection. The above findings suggested that LP postbiotics, particularly the metabolites, were superior to the active probiotic in rescuing ST-induced anxiety-like behaviors in mice.

### The LP postbiotics, particularly the metabolites, prevent ST-induced cognitive impairment and depressive-like behaviors in mice

**Figure 7:**
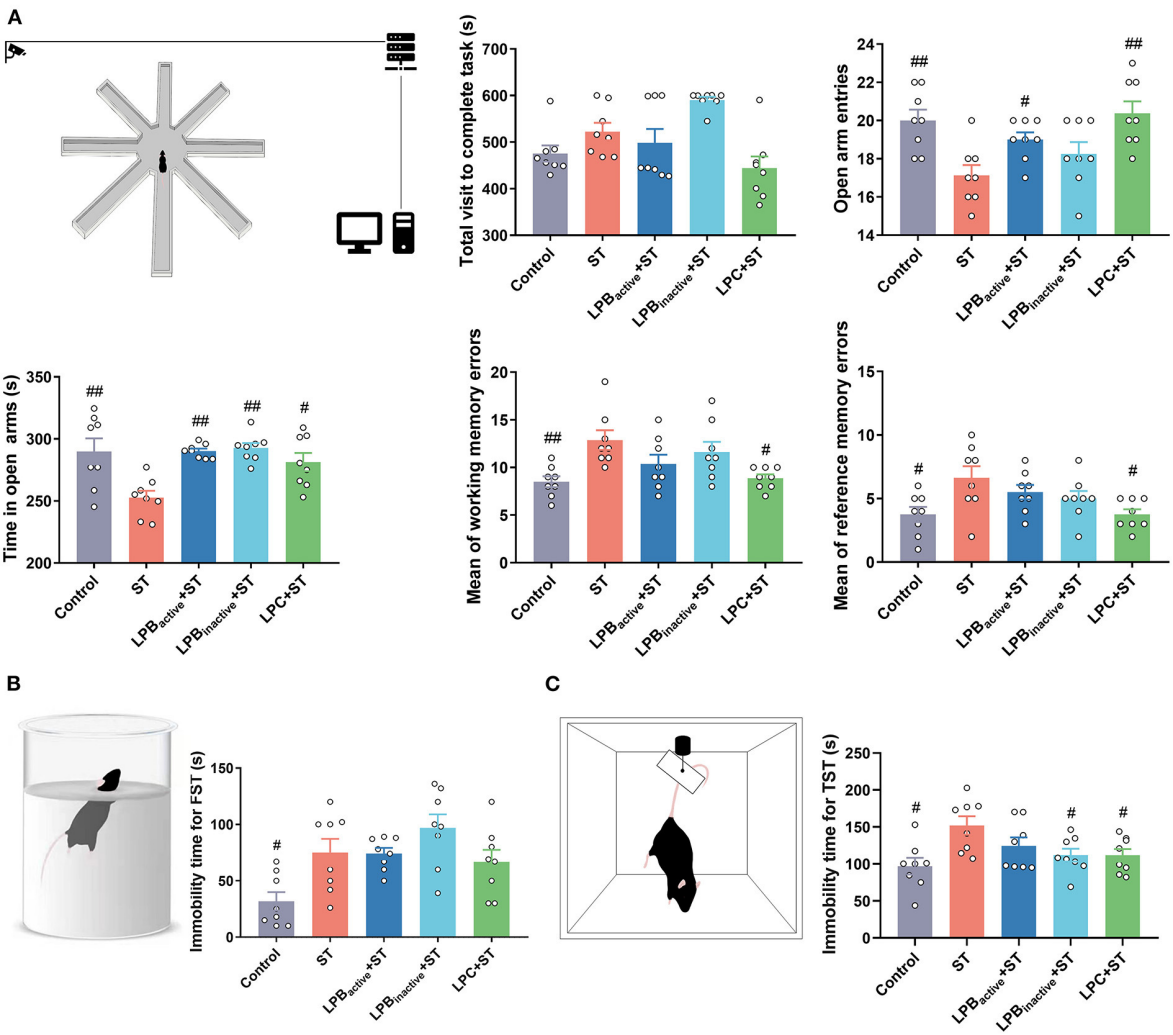
Radial arm maze (RAM), forced swim test (FST), and tail suspension test (TST) representing cognitive impairment and depressive-like behaviors. **(A)** RAM data. A cartoon of the apparatus for RAM is shown. Statistical analysis of the total visit to complete task, open arm entries, time in open arms, and mean of working and reference memory errors. **(B)** FST data. A cartoon of the apparatus for FST and the analysis of immobility time. **(C)** TST data. A cartoon of the apparatus for TST and the analysis of immobility time. The data shown as mean ± SEM were analyzed by one-way ANOVA and Tukey's test (*N* = 8 in each group). ^#^Indicates the significance compared to ST group. ^#^*p* < 0.05, ^##^*p* < 0.01.

As shown in Figure 7A, the RAM test presenting learning and memory ability showed that ST infection dramatically reduced the number of open arm entries (*p* < 0.01), whereas LPC as well as LPB_active_ administrations markedly increased it (*p* < 0.05, *p* < 0.01, respectively). Similarly, the time spent in the open arm was decreased by ST challenge (*p* < 0.01), but the trend was significantly reversed by LPB_active_, LPB_inactive_, and LPC (*p* < 0.01, *p* < 0.01, *p* < 0.05, respectively). The ST infection significantly increased the mean of working and reference memory errors (*p* < 0.01 and *p* < 0.05, respectively), but were markedly altered by LPC pretreatment (*p* < 0.05). The performance in FST and TST reflect the depressive-like behaviors. The FST results showed that ST markedly increased the immobility time (*p* < 0.05), and LPC could reverse it to some degree although the difference was not significant (Figure 7B). The immobility time for TST was significantly increased (*p* < 0.05), but was markedly decreased by LP postbiotic pretreatments (*p* < 0.05) (Figure 7C). These results showed that LP postbiotics, especially the metabolites, could suppress ST-induced cognitive impairment and depression and even had more advantages than the live probiotic.

### The LP postbiotics exhibit better effects than the active bacteria in modulating levels of neuroactive molecules in mice

**Figure 8:**
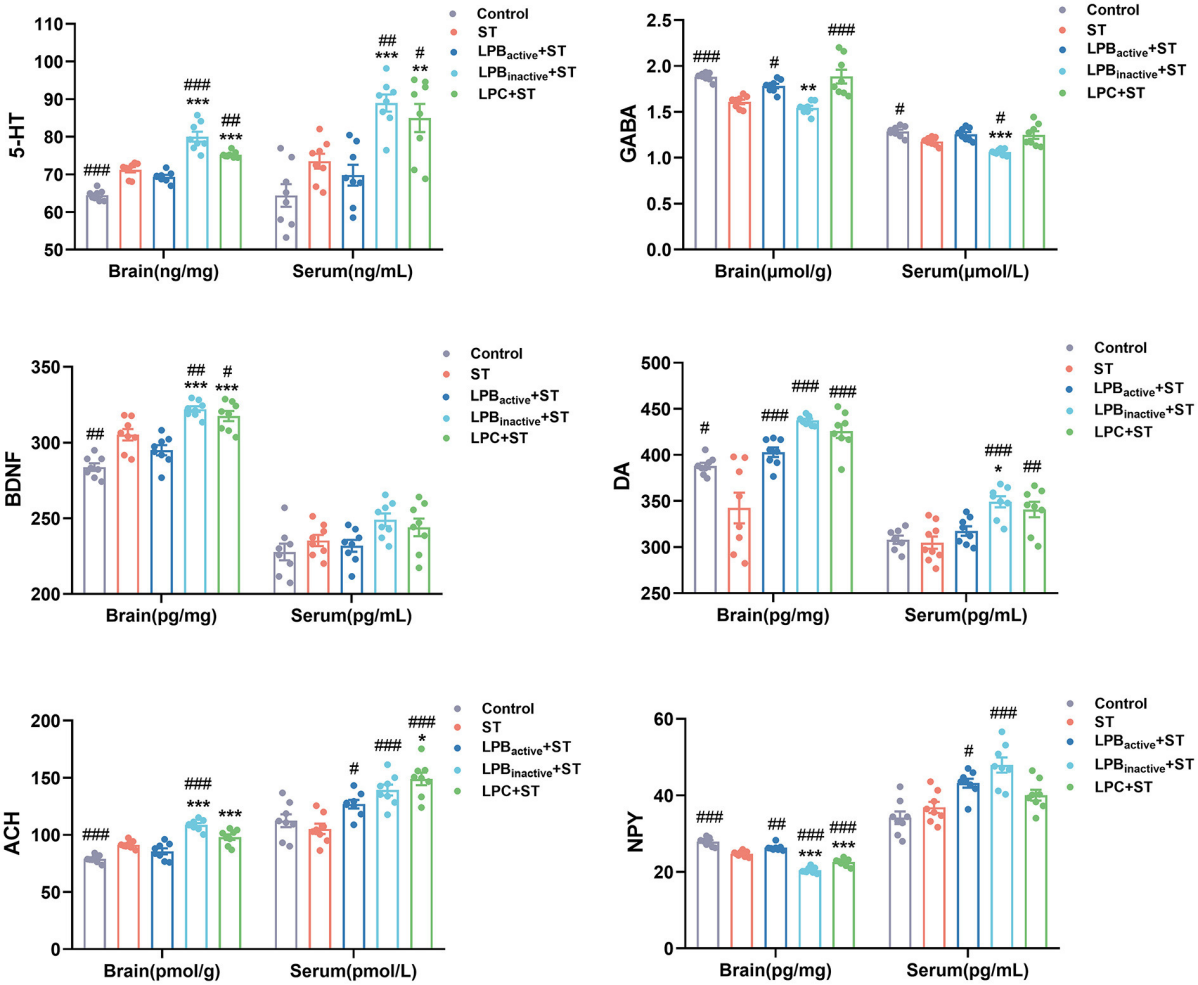
The LP postbiotics modulate the levels of neuroactive molecules in mice. Concentrations of neuroactive molecules (5-HT, GABA, BDNF, DA, ACH, and NPY) in brain and serum. The data shown as mean ± SEM were analyzed by one-way ANOVA and Tukey's test (*N* = 8 in each group). ^#^Indicates the significance compared to ST group: ^#^*p* < 0.05, ^##^*p* < 0.01, ^###^*p* < 0.001. *Indicates the significance compared to LPB_active_ + ST group: *p < 0.05, **p < 0.01, ***p < 0.001.

We then further detected the levels of neuroactive molecules in murine brain and serum (Figure 8). The results showed that the concentration of 5-HT was markedly increased in LP postbiotics pretreated groups when compared to the ST group both in the brain and in the serum (*p* < 0.00 and *p* < 0.01, respectively; *p* < 0.01 and *p* < 0.05, respectively), whereas the active bacteria showed no significant difference (*p* > 0.05). The ST challenge significantly downregulated the level of brain GABA (*p* < 0.001), but this trend could be altered by LPB_active_ and LPC intervention (*p* < 0.05 and *p* < 0.001, respectively). The ST also decreased serum GABA (*p* < 0.05), whereas LP pretreatments could not inhibit this reduction (*p* > 0.05). The brain BDNF level was notably increased by LPB_inactive_ and LPC pretreatments (*p* < 0.01 and *p* < 0.05, respectively), while LPB_active_ showed no effects (*p* > 0.05). No obvious changes were found in serum BDNF among groups (*p* > 0.05). ST infection dramatically decreased the level of brain DA, while LP pretreatments could notably reverse this trend (*p* < 0.001); a marked upregulation was also found by LPB_inactive_ and LPC pretreatments (*p* < 0.001 and *p* < 0.01, respectively). Compared to the ST group, LPB_inactive_ markedly increased the ACH level both in the brain and serum (*p* < 0.001), and LPB_active_ and LPC significantly increased the serum ACH (*p* < 0.05 and *p* < 0.001, respectively). The concentration of brain NPY was reduced by ST challenge (*p* < 0.001), LPB_active_ pretreatment reversed it to the normal level (*p* < 0.01), while LPB_inactive_ and LPC showed a an opposite effect (*p* < 0.001). In the serum, LPB_active_ and LPB_inactive_ significantly increased the NPY level (*p* < 0.0 and *p* < 0.001, respectively). Interestingly, the LP postbiotic pretreatments showed a stronger capacity in increasing the level of 5-HT in both brain and serum; brain BDNF; and brain ACH (*p* < 0.001), when compared to the active bacteria pretreatment. Moreover, LPB_inactive_ and LPC pretreatments exhibited an opposite effect for serum NPY. The results indicated that LP could modulate the neuroactive molecules in mice, and its postbiotics present better effects than the live probiotic.

### The LP postbiotics modulate the production of SCFAs

**Figure 9:**
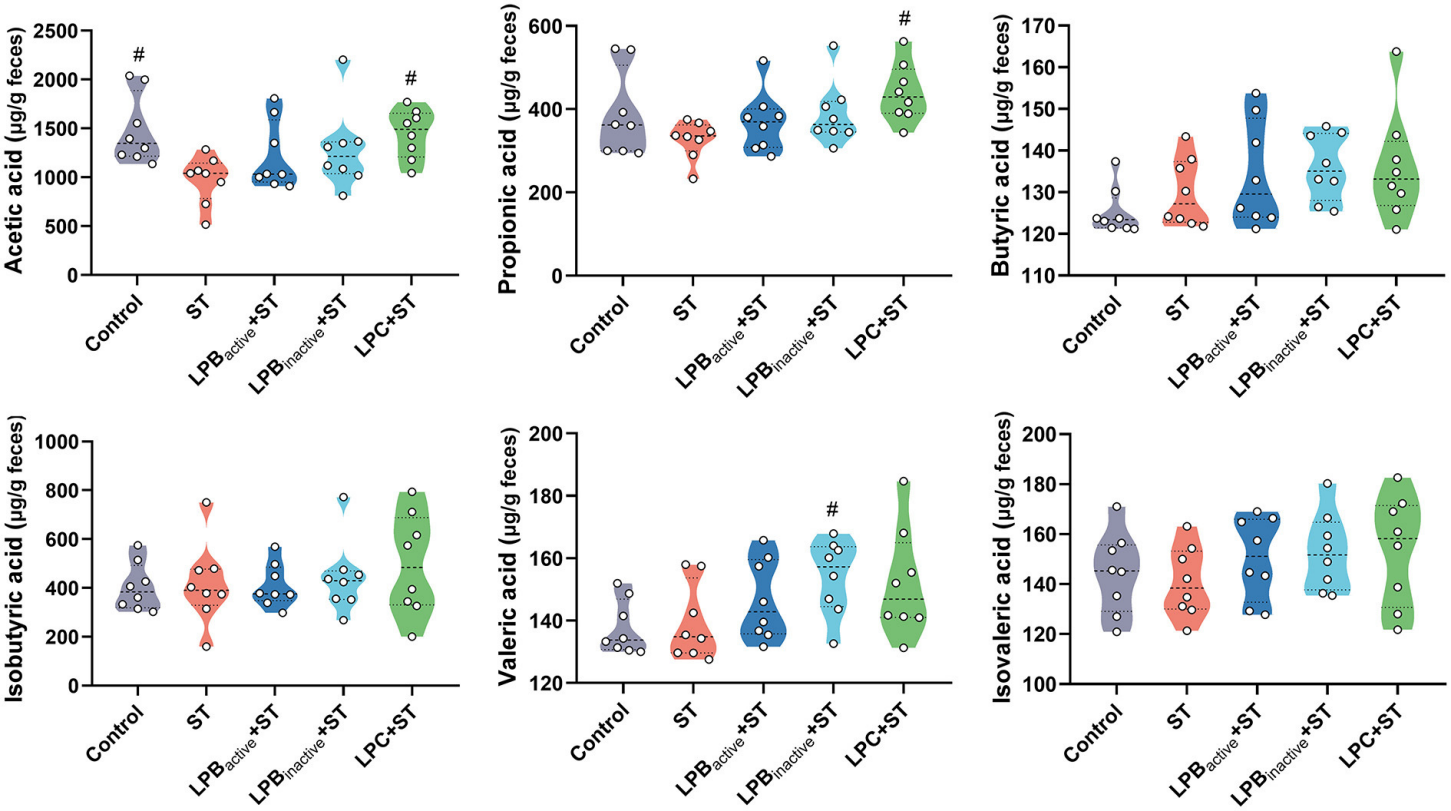
Violin plots displaying the concentrations of short-chained fatty acids (SCFAs) of cecal microbiota. The data shown as mean ± SEM were analyzed by one-way ANOVA and Tukey's test (*N* = 8 in each group). ^#^Indicates the significance compared to ST group: ^#^*p* < 0.05.

As displayed in Figure 9, the ST challenge significantly decreased the concentration of acetic acid (*p* < 0.05), while the LPC pretreatment could markedly increase it (*p* < 0.05). The LPC also notably upregulated the level of propionic acid in comparison to that in the only ST-infected mice (*p* < 0.05). Moreover, there was a significant increase of valerate acid in LPB_inactive_+ ST group (*p* < 0.05). No significant differences in butyrate acid, isobutyric acid, and isovaleric acid were observed in response to the ST and LP treatments (*p* > 0.05).

### The LP postbiotics, particularly the metabolites, optimize the composition of cecal microbiota

**Figure 10:**
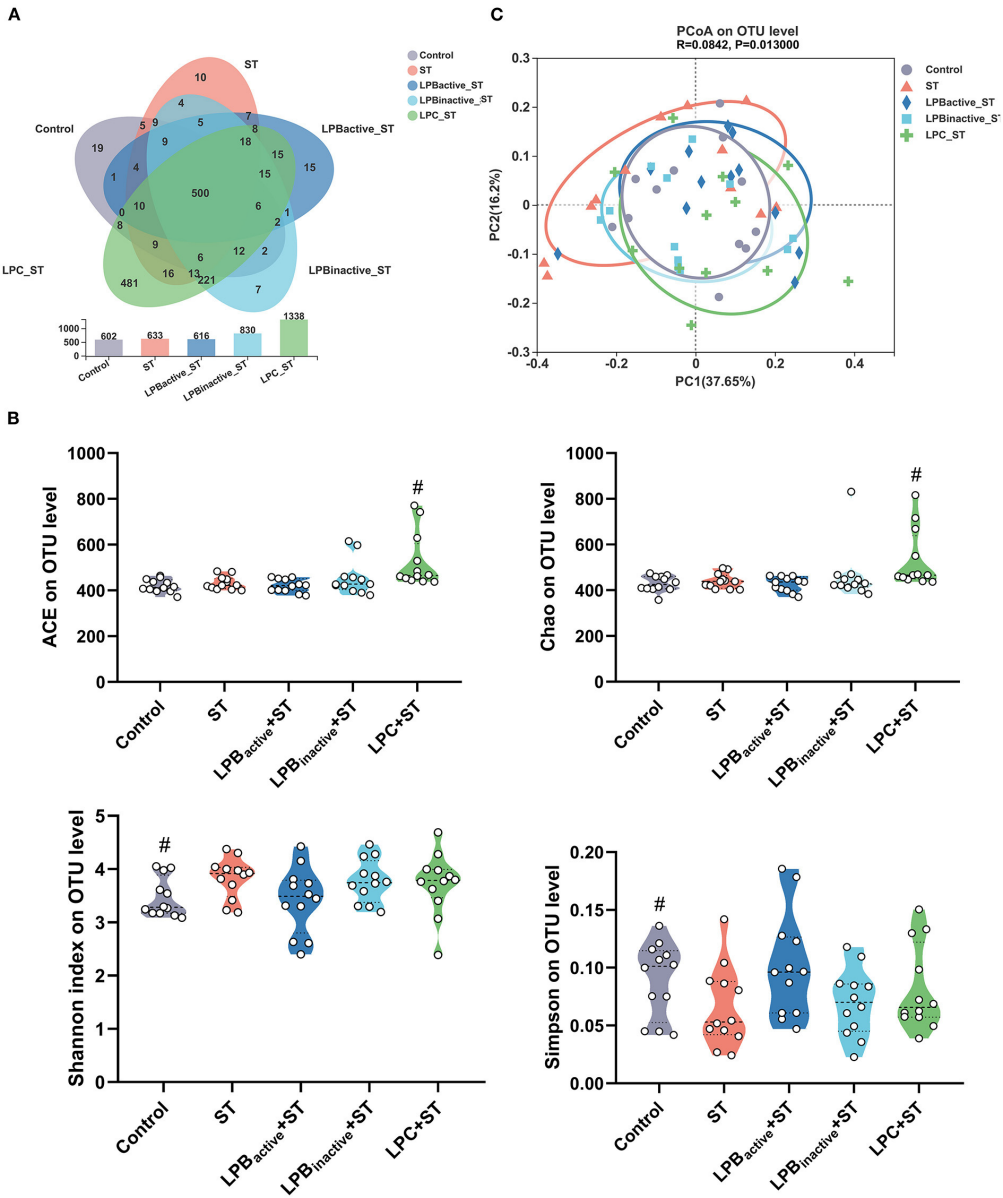
Analysis of the diversity of gut microbiota. **(A)** Venn diagram indicating the operational taxonomic units (OTUs) in each group. **(B)** The indexes representing α diversity at the OTU level. **(C)** The β-diversity displayed in a principal component analysis (PCA) scatterplot. The data shown as mean ± SEM were analyzed by one-way ANOVA and Tukey's test (*N* = 12 in each group). ^#^Indicates the significance compared to ST group: ^#^*p* < 0.05.

We then investigated the alterations in the cecal microbiota during ST challenge. The total OTUs in the Control, ST, LPB_active_ + ST, LPB_inactive_ + ST, and LPC + ST were 602, 633, 616, 830, and 1,338, respectively, and the LPC pretreatment showed markedly higher unique OTUs (481) than the other two groups (Figure 10A). Figure 10B displays the parameters representing α diversity. The LPC significantly increased ACE and Chao indexes that indicate microbiota richness. The ST challenge markedly decreased Shannon and increased Simpson indexes presenting microbiota richness. The PCA scatterplot indicating β diversity revealed an obvious shift of the Control and ST groups, whereas all the LP-pretreated groups could reverse this trend to the normal (Figure 10C).

**Figure 11:**
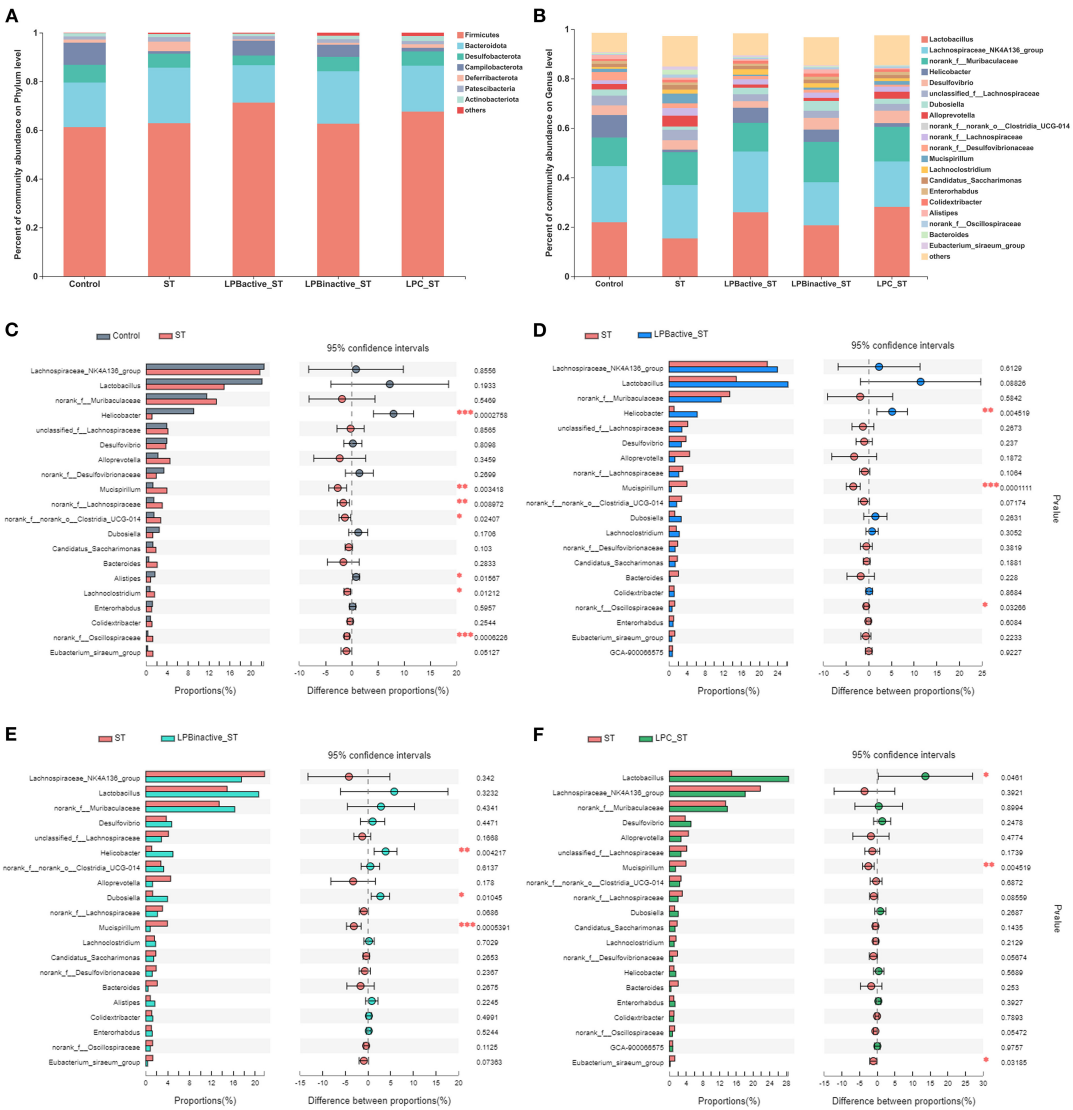
The LP postbiotics optimized the composition of cecal microbiota. **(A)** Bar graph of microbiota composition at the phylum level. **(B)** Bar graph of microbiota composition at the genus level. **(C–F)** Comparisons of gut microbiota (Control vs. ST, ST vs. LPB_active_ + ST, ST vs. LPB_inactive_ +ST, ST vs. LPC + ST) at genus level by statistical analysis of taxonomic and functional profiles (STAMP) (*N* = 12 in each group). The data were analyzed by Student's *t*-test (*N* = 12 in each group). *p < 0.05, **p < 0.01, ***p < 0.001.

Figure 11 presents the differences in microbial compositions at the phylum and genus levels. As shown in Figure 11A, ST challenge obviously decreased Campilobacterota and increased Defferibacterota, but the LP pretreatments could reverse this trend. Figure 11B indicated there were variations of microbiota composition at genus level. The specific changes were displayed in Figures 11C–F by STAMP analysis of the TOP20 genera. Compared to the Control group, ST challenge markedly decreased *Helicobater* (*p* < 0.001) and *Alistipes* (*p* < 0.05), and increased *Mucispirillum* (*p* < 0.01), norank_f_Lachnospiraceae (*p* < 0.001), norank_f_norank_o_ Clostridia_UCG-014 (*p* < 0.05), *Lachnoclostridium* (*p* < 0.05), and norank_f_Oscillospiraceae (*p* < 0.001) (Figure 11C). In comparison to ST group, the proportion of *Helicobacter* was notably increased (*p* < 0.01), while *Mucispirillum* and norank_f_Oscillospiraceae were decreased (*p* < 0.001 and *p* < 0.05, respectively) in LPB_active_ + ST group (Figure 11D). LPB_inactive_ pretreatment significantly upregulated the abundance of *Helcobacter* and *Dubosiella* (*p* < 0.001 and *p* < 0.05, respectively), and downregulated *Mucispirillum* (*p* < 0.001) (Figure 11E). The LPC showed a strong capacity to increase the richness of *Lactobacillus* (*p* < 0.05), and decrease the abundance of *Mucispirillum* and *Eubacterium_siraeum*_group (*p* < 0.05) (Figure 11F).

**Figure 12:**
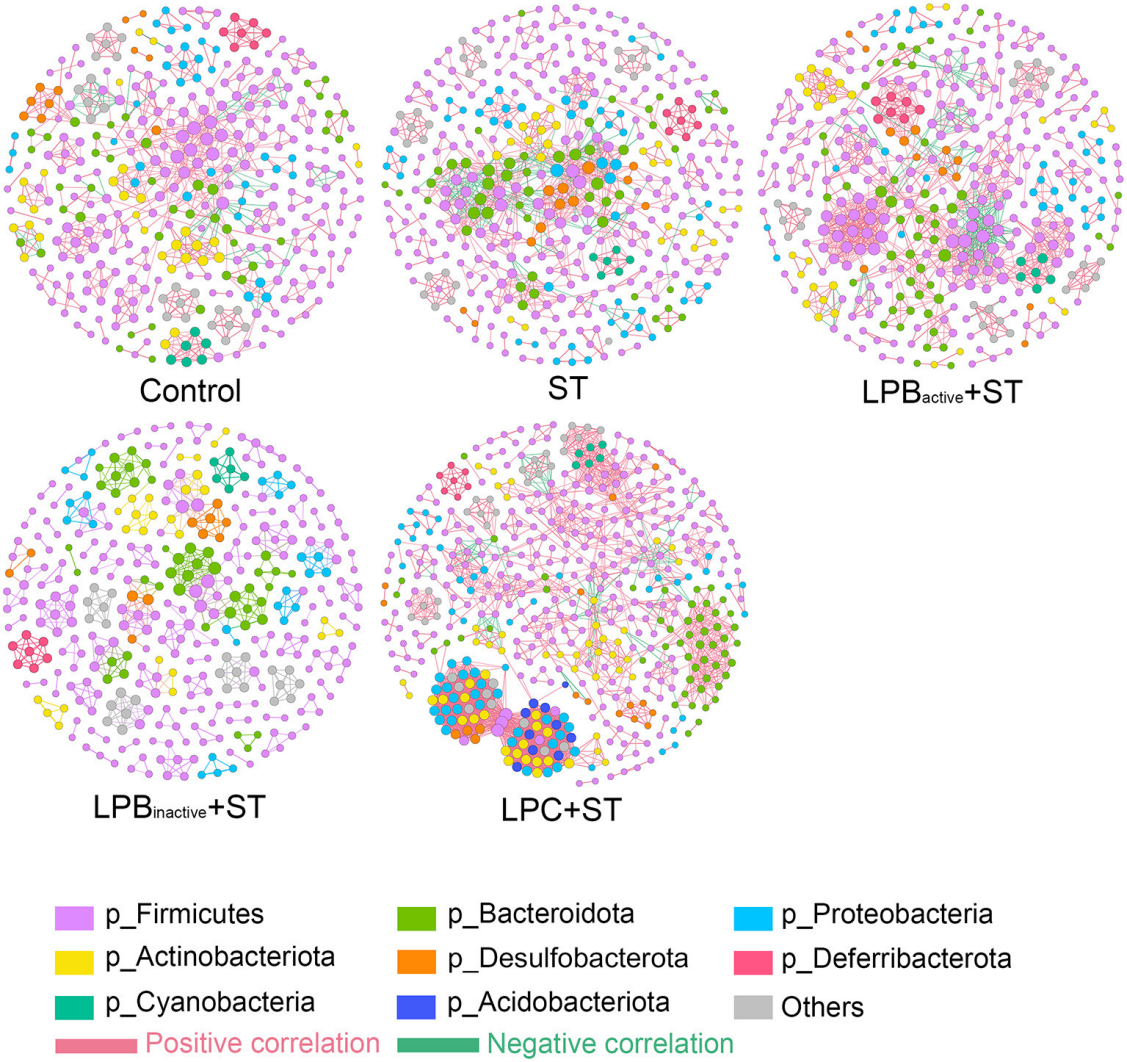
Co-occurrence networks of microbial communities. A connection represents a very strong (Spearman's R > 0.6) and significant (FDR-adjusted *p* < 0.05) correlation. The size of each node is proportional to the relative abundance; the thickness of each connection (edges) between two nodes is proportional to the value of Spearman's correlation coefficients. Red and green lines, respectively indicate the positive and negative correlations.

As for the co-occurrence network displayed in Table 1 and Figure 12, results showed that the number of nodes and edges in LPC + ST group was obviously higher than the other groups. The LPB_active_ and LPC pretreatments increased the average degree when compared with ST group, while LPB_inactive_ decreased it. Similar results were also found in network diameter and graph density. The modularity values in all groups were > 0.4; ST challenge, as well as LPB_active_ and LPC pretreatments downregulated it, whereas LPB_inactive_ showed an opposite trend, when compared to the Control group. The positive correlation among the microbes was decreased by ST challenge, while LP postbiotics, particularly LPC could reverse this trend. On the contrary, ST increased the negative correlation, whereas LPC administration apparently decreased it.

**Table 1:** Topological properties of co-occurrence network.

	**Control**	**ST**	**LPB**_active_ + **ST**	**LPB**_inactive_ + **ST**	**LPC** + **ST**
Nodes	367	404	392	371	501
Edges	802	1,007	1,264	698	2,799
Average degree (AD)	4.371	4.985	6.449	3.763	11.174
Network diameter (ND)	14	14	14	8	21
Graph density (GD)	0.012	0.012	0.016	0.01	0.022
Modularity (MD)	0.898	0.786	0.828	0.95	0.771
Positive correlation	90.52%	85.30%	87.50%	92.84%	96.36%
Negative correlation	9.48%	14.70%	12.50%	7.16%	3.64%

### Correlations between phenotypic variables and microbial communities

**Figure 13:**
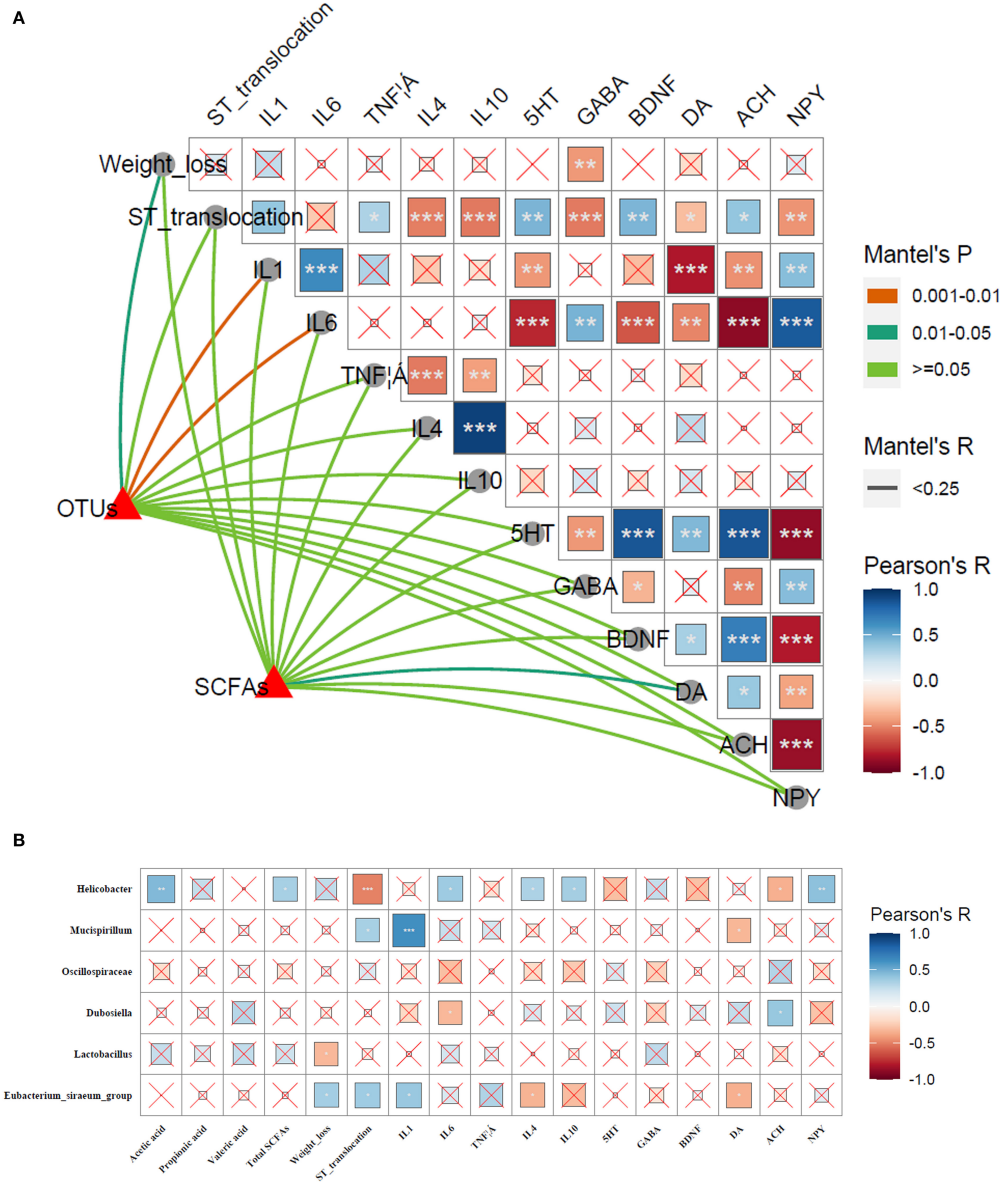
Correlations between phenotypic variables and microbial communities. **(A)** The relationship between phenotypic variables and microbial communities. The Pearson correlation coefficient denotes Pairwise comparisons of phenotypic variables with a color gradient. Taxonomic structures and SCFAs were linked to each phenotypic variable by Mantel correlation based on Bray–Curtis dissimilarity. The line thickness indicates the Mantel's R statistic for the corresponding distance correlations, and the color indicates statistical significance. *p < 0.05; **p < 0.01; ***p < 0.001. **(B)** Pearson's correlation analysis. Correlations of phenotypic variables (SCFAs, weight loss, ST translocation in brain, inflammatory cytokines, and neuroactive molecules) and microbial communities. The color and the dot size represent the correlation coefficient. *p < 0.05; **p < 0.01; ***p < 0.001.

The correlations among microbial communities (microbial structure, SCFAs) and phenotypic variables (weight loss, ST translocation in brain, inflammatory cytokines, and neuroactive molecules) were conducted to further clarify the participance of gut–brain axis in alleviating *Salmonella*-induced neurological dysfunctions. As shown in Figure 13A, shifts in microbial communities and SCFAs were tightly linked to phenotypic variables, as revealed by the Mantel test. To be specific, the OTUs were strongly linked to weight loss (*p* < 0.05), IL-1β, and IL-6 (*p* < 0.01), whereas SCFAs showed a significant correlation to DA. Pearson correlation analysis revealed that the weight loss was negatively linked to GABA (*p* < 0.01). The IL-1β showed markedly negative relationships to 5-HT (*p* < 0.01), DA (*p* < 0.001), and ACH (*p* < 0.001), and a positive link to NPY (*p* < 0.001); IL-6 was positively correlated to GABA (*p* < 0.01) and NPY (*p* < 0.001), and was notably negative to 5-HT (*p* < 0.001) and BDNF (*p* < 0.001), DA (*p* < 0.01), and ACH (*p* < 0.001). The DA exhibited a negative relationship to the ST translocation in brain, IL-1β and IL-6 (*p* < 0.05, *p* < 0.001, and *p* < 0.01, respectively), and a positive link to 5-HT and BDNF (*p* < 0.01 and *p* < 0.05, respectively).

To further investigate the specific relationships between significant microbes changed by LP and phenotypic variables, the Pearson's correlation analysis was performed (Figure 13B). The results revealed that *Helicobacter* was significantly positive to acetic acid (*p* < 0.01), total SCFAs (*p* < 0.05), IL-6 (*p* < 0.05), IL-4 (*p* < 0.05), IL-10 (*p* < 0.05), and NPY (*p* < 0.01), and negative to ST translocation in brain and ACH (*p* < 0.001 and *p* < 0.05, respectively). *Mucispirillum* showed a positive link to ST translocation in brain and IL-1β (*p* < 0.05 and *p* < 0.001, respectively), and negative to DA (*p* < 0.05). *Dubosiella* was negatively related to IL-6 and positive to ACH (*p* < 0.05). A significant negative correlation was found between *Lactobacillus* and weight loss (*p* < 0.05). *Eubacterium_siraeum*_group exhibited a strongly positive correlation with weight loss, ST translocation in brain and IL-1β (*p* < 0.05), and was negatively linked to IL-4 and DA (*p* < 0.05).

## Discussion

The relationship between probiotics and improved systemic health has attracted considerable scientific interests for decades. Probiotics exert numerous benefits including pathogen inhibition, metabolism management, immune modulation and disease prevention (29). However, as probiotics are live microorganisms which are difficult to survive in extreme conditions, and have potential resistance genes and virulence factors (21), exploring the effects of probiotics' components (postbiotics) have become a research hotspot. Studies have found that postbiotics performed equivalently or even better to the active bacteria. In this study, we demonstrated that LP postbiotics, particularly the metabolites, exhibited a stronger capacity in alleviating *Salmonella*-induced injuries, as evidenced by the significant inhibition of weight loss, suppression of ST translocation, and alleviation of tissue injuries and neurological dysfunctions. Consistently with our findings, it was reported that *Lactobacillus*-derived postbiotics showed immunomodulatory and protective capacity against *Salmonella* infection in mice (30).

The LP postbiotics protected mice against *Salmonella*-induced systemic injuries. LPB_inactive_ as well as LPB_active_ administration increased mice weight in comparison to the Control group, but LPC showed an opposite trend. These results revealed the different efficacy of probiotic components. The reason for the unexpectedly reduced trend of weight ratio from Day 9 to 12 might be due to the stimuli and the changed environment caused by the 1-week training for RAM test before ST challenge. Rapid weight loss is a clinical symptom of *Salmonella* infection which is due to the decreased feed intake, gastrointestinal disorders and abnormal physiological state (32). It was reported that administration of probiotics such as *Saccharomyces boulardii* and *Lactobacillus acidophilus* could prevent weight loss caused by *Salmonella* challenge (33, 34). Differently, we found that pretreatment of LP metabolites markedly inhibited weight loss whereas LP bacteria showed no differences, suggesting the superiority of LP metabolites in preventing *Salmonella* infection. The possible reasons might be that the metabolites contain many antibacterial substances including organic acids and bacteriocin which directly inhibit pathogen growth (35); furthermore, some metabolites such as SCFAs and exopolysaccharides bind to the receptors in intestinal epithelial cells can enhance cell immunity (36), contributing to responding rapidly upon *Salmonella* infection. However, live bacteria compete for colonization sites with commensal bacteria, which might disturb micro-ecology balance and exert a side effect to the host. Similar with our results, it was reported that the metabolites of lactic acid bacteria exerted a better effect than their live probiotics on inhibiting porcine epidemic diarrhea virus (37). The above results further confirmed the importance of targeting the specific components for probiotic application. As the invasive bacteria, *Salmonella* is capable of crossing the intestinal mucosa, transporting through the bloodstream and cause tissue injuries. *Salmonella* is believed to colonize the colon and damage colonic mucosa, and the shortening of colon lengths normally indicates the severity degree of infection (38). Therefore, the longer colon length by LP pretreatments suggesting the decreased infection. Spleen is another main targeted organ. *Salmonella* commonly induces prolonged splenomegaly in mice due to the massive splenic erythropoiesis (39). In his study, a marked increase in spleen size was found in ST group whereas LP pretreatment could inhibit it, suggesting the alleviated injuries. Similarly, it was reported that *Bacillus amyloliquefaciens* significantly decreased the splenomegaly and spleen index caused by ST infection (40).

The LP postbiotics markedly inhibited *Salmonella*-induced brain injury and neuroinflammation. Although the exact mechanism mediating host brain invasion remains obscure, one study highlighted the vital role of its outer membrane protein A and SPI-1 genes in this process (9). The reduced ST translocation suggested the capacity of LP postbiotics in pathogen clearance and ameliorating brain injuries. Our results also showed that LP postbiotics were capable of suppressing neuroinflammation. Neuroinflammation caused by *Salmonella* has been reported by many studies. For instance, ST infection could recruit the inflammatory monocytes to the CNS in mice (41); *Salmonella*-derived LPS markedly activated the expression of TNF-α, IL-1β, and IL-6 in microglia and astrocytes *in vitro* (42). IL-1β and IL-6 are pro-inflammatory cytokines that can damage CNS cells, and induce acute neurodegenerative pathology (43). Conversely, as the main anti-inflammatory cytokines, IL-4 and IL-10 counteract the inflammatory responses caused by stimuli and relieve CNS damage (44). Thus, the decreased IL-1β and IL-6, and increased IL-4 and IL-10 indicated the effectiveness of LP postbiotics on suppressing *Salmonella*-induced neuroinflammation. Surprisingly, LP postbiotics performed better than active bacteria in reversing *Salmonella*-induced inflammatory responses. We speculate that as the live probiotics are immune activator, and it shows a greater potential to trigger inflammatory response; however, the inactive bacteria and metabolites drive functions relying on the chemical components which exhibit milder effects. This view can be supported by one study that *L. casei* postbiotics were more pronounced than the live bacteria in reducing LPS-activated pro-inflammatory cytokines and increasing IL-10 expression in an *ex-vivo* organ (45). Therefore, the above results confirmed the effectiveness and superiority of LP postbiotics in protecting against the infection-induced brain injuries and neuroinflammation.

The LP postbiotics strongly prevented *Salmonella*-induced behavior disorders in mice. LDB and OFT are the most popular ethological tests to assess anxiety-like behaviors. In LDB test, anxiety is reflected by the conflict between the tendency to explore and avoid unfamiliar environment, which is evaluated by the decreased number of transitions and time spent in the light chamber (46). In this study, LP postbiotics markedly reversed the reduction of LDB transitions, time spent in the light chamber and number of central entries caused by ST challenge, proving the anxiolytic-like property of LP postbiotics. As for OFT, the anxiety level is determined with periphery and central time/ entries ratio, and increases in center activity are indicative of anxiety relief (47). We found that pretreated with LPC exhibited a notable increase in the indices reflecting anxiolytic effects. Our results highlighted the superiority of LP metabolites in suppressing infection-induced anxiety. Consistently, *Lactobacillus reuteri* has been showed to attenuate anxiety-like behavior by OFT test during *C. rodentium* infection (48). In addition, the cognitive impairment and depression-like behaviors in mice during *Salmonella* infection were evaluated by RAM, FST, and TST. The RAM results revealed that ST challenge dramatically decreased open arm entries and the time spent in open arms, and increased the mean of working and reference memory errors, whereas LP pretreatments, particularly LPC could reverse this trend. Our results suggested the capacity of LP in reducing ST-mediated spatial memory impairment. Similar to our results, it was reported that supplementation of *Lactobacillus* and *Bifidobacterium* mixtures could attenuate hippocampus injury and learning and memory impairments in mice (49). Depression is one of the main diagnosed neuropsychiatric disorders. Depression-like behavior tested by FST and TST are based on the assumption that the animal will try to escape the stressful situation, and the duration of immobility indicates the degree of behavioral responses to depressant-like activity (50). We found that LPB_inactive_ and LPC significantly inhibited the increased immobility time by ST infection both in FST and TST, indicating its role in anti-depression. This effect has been confirmed by one study that oral probiotics ameliorated the depressive-like behaviors induced by chronic mild stress in mice (51). Taken together, our findings demonstrated that LP postbiotics, particularly the metabolites exhibited a protective role in suppressing *Salmonella-*induced behavior deficits.

Neuroactive molecules including transmitters, neurotrophic factors and neuropeptides play a crucial role in driving neural functions and behaviors. In this study, LP markedly modulated their levels in *Salmonella*-infected mice, and surprisingly the postbiotics exhibited better effects than the active probiotic. 5-HT is a key neurotransmitter involving in emotional control, food intake, pain processing and enteric physiological functions (52). We found a high concentration of 5-HT both in brain and serum by LP postbiotic pretreatments. It was reported that over 90% of 5-HT is produced in intestine, and gut microbiota takes an important part in this process. Consistently with our findings, studies revealed that heat-killed *L. casei* and the metabolites of gut microbes could promote colonic 5-HT synthesis (53, 54). Moreover, GABA is a primary inhibitory neurotransmitter which is vital to protect against behavior disorders including anxiety, depression, and stress (55). This study revealed that *Salmonella* infection markedly decreased GABA levels while LPB_active_ and LPC pretreatments were associated with a return to control levels. Accumulating evidence has confirmed the ability of probiotics to directly produce or trigger the host to produce GABA through the conversion of amino acid glutamate (20). The increased GABA is essential to alleviate anxiety- and depressive-like behaviors (55). Furthermore, it was reported that GABAergic activation can enhance host antimicrobial responses to intracellular bacterial infection (56), which further proved the protective effects of LP postbiotics by upregulating GABA during *Salmonella* infection. The DA is a neurotransmitter which not only regulates behavior and movement but also as a key molecule bridging the immune and nervous systems. Many studies have highlighted its strong ability to suppress neuroinflammation (57). In this study, LP pretreatments significantly upregulated the levels of DA that was decreased by *Salmonella*, suggesting its role in protecting against infection-induced inflammation. The BDNF is a member of the neurotrophin family that is pivotal in the differentiation, survival and protection of neurons (15). Studies showed that chronic infection can lead to the reduced BDNF expression and increased anxiety-like behaviors (41). Differently, we found that *Salmonella* significantly increased the expression of BDNF. This might be due to that in this study *Salmonella* infection was acute, which might be supported by that some stimuli such as stress and hypoxia activate the expression of BDNF (58). Consistently with our results, it was reported that maternal acute exposure with LPS markedly increased the levels of BDNF in fetal and neonatal brain (59). Similar reason could also explain the expression of ACH. The NPY is a key neuropeptide that regulates immune functions and allows for communication between the immune and nervous systems (60). Also, NPY plays an important role in controlling inflammatory processes and stress. In this study, the increased NPY by LP pretreatments might participate in inhibiting systemic inflammation, whereas the decreased levels in brain suggested the suppressed immune responses and alleviated brain dysfunctions. The above results revealed that LP postbiotics modulated the expression of neuroactive molecules, which contributed to alleviating the behavior disorders and inflammation during *Salmonella* challenge.

As the main part of gut–brain axis, the gut microbiota is a biological barrier against pathogen infection, and its alterations play a pivotal role in modulating brain functions (61). In this study, LP postbiotics, particularly the metabolites showed a strong ability to alter microbiota during *Salmonella* infection. LPC markedly increased the total OTUs and α-diversity when compared to ST group, indicating its capacity in improving the abundance of gut microbiota. The mechanism might be that the culture supernatant of *L. plantarum* contains various metabolites such as lactic acid, SCFAs, polysaccharides, and enzymes (62), which are beneficial for commensal bacteria growth. At the genus level, ST infection caused a marked decrease of *Helicobacter* and a significant increase of *Mucispirillum*, while LP pretreatments could markedly reverse this trend; a reduction of *Lactobacillus* was also found in ST group, but this trend was markedly reversed by LP administration, particularly LPC. *Helicobacter* is a Gram-negative and microaerophilic genus that is commonly colonized in the gastrointestine, and some species are believed to be detrimental for the host which may cause chronic inflammation, ulcer, and even cancer (63). However, this study showed that *Salmonella* significantly reduced its abundance, whereas LPB pretreatments reversed it to the normal. The possible reason might be that bacterial strains from even same species might exert opposite effects. For instance, *Escherichia coli* strain Nissle 1917 is a probiotic, while others are commonly regarded as pathogens (64). Nevertheless, the underlying mechanism is needed to be further investigated. *Mucispirillum*, a genus in the phylum Deferribacteres, is Gram-negative, obligate anaerobic, flagellated and able to move through mucus (65). Numerous studies have revealed that the increase of *Mucispirillum* spp. is highly linked to obesity, infection, inflammatory bowel disease and stress (66, 67). For instance, a study showed that *Mucispirillum schaedleri* triggered spontaneous colitis and was causally linked to the development of Crohn's disease in immunodeficient mice (68). We found that ST increased the abundance of *Mucispirillum* but LP postbiotic pretreatments could markedly decrease it, suggesting the alleviated infection and intestinal disorders. *Lactobacillus* is a well-known probiotic in the gut, which exerted various benefits to the host including inhibiting pathogens and inflammation (69). The view that pathogen infection influences the richness of *Lactobacillus* have been supported by many studies (70, 71). This study showed that LPC reversed the reduction of *Lactobacillus* caused by ST challenge, indicating the optimized microbiota. Surprisingly, LP metabolites exhibited a better effect than its active bacteria. The supernatants of *L. plantarum* contain various organic acids such as lactic acid, phenyllactic acid, hydroxyphenyllactic acid, and indole lactic acid (72), which exhibit strong capacity to inhibit pathogenic bacteria but promote the growth of acid-tolerant probiotics. Therefore, we speculate that this might explain the possible mechanism of the increased abundance of *Lactobacillus* by LPC pretreatment. The findings further prove the superior of postbiotic than its active bacteria. In addition, our results revealed that LPC pretreatment exhibited more obvious changes of the co-occurrence network, which provide insight into microbial interactions. We found that the topological parameters including nodes, edges, average degree and network diameter of microbial network in LPC + ST group was apparently higher than those of other groups. These illustrate that LPC shows stronger ability to increase microbe connections. The modularity values were more than 0.4 in all groups, revealing a modular structure of the network (73). All the findings demonstrated that LP postbiotics, particularly the metabolites could optimize the gut microbiota.

Finally, the correlation analysis further confirmed a strong relationship between LP postbiotics-mediated neurological dysfunction alleviation and the gut microbiota. We found that *Lactobacillus* was strongly negative to weight loss. Weight loss is a key parameter indicating the severity of infection. Many studies have proved that *Salmonella* infection causes a dramatic reduction of weight (33). We found that ST markedly decreased weight loss and the abundance of *Lactobacillus*, but LPC pretreatment could notably reverse this trend. Thus, the strongly negative link between these two indexes indicated that the increased *Lactobacillus* by LP pretreatments played a pivotal role in alleviating ST infection. These finding are in line with those by (74) who found that oral administration of *Lactobacillus* prevented body weight losses after virus infection. Furthermore, the gut microbiota exhibited a marked correlation with the inflammatory cytokines. *Helicobacter* was positive to IL-4 and IL-10 and it could be markedly increased by LPB_active_ and LPB_inactive_ pretreatments, suggesting LPB might alleviate inflammation by increasing the abundance of *Helicobacter*. Differently, this genus was generally regarded as inflammatory-promoting bacteria by the previous studies (75), and we speculated this might be due to the specific strain. This study first demonstrated the beneficial effects of *Helicobacter* during *Salmonella* infection. We also found a strongly positive correlation between *Mucispirillum* and IL-1β, and LP evidently suppressed the increase of this genus caused by ST challenge. This result revealed that *Mucispirillum* took an important part in ST-induced neuroinflammation. Consistently, the pro-inflammatory effect of *Mucispirillum* has been proved by a study that the abundance of *Mucispirillum* was associated with the increased risk of inflammatory bowel disease (76). *Eubacterium_siraeum*_group showed a positive link with IL-1β and a negative link with IL-4, indicating its pro-inflammatory effects. It was reported that *Eubacterium siraeum* exhibited a capacity to drive gut barrier dysfunction and intestinal inflammation (77). The decreased of this genus by LPC pretreatment indicated an inhibited inflammatory level in mice brain. Therefore, the findings suggested that LP postbiotic pretreatments modulated the composition of microbiota to alleviate *Salmonella*-induced neuroinflammation. Furthermore, DA was negatively linked to the proinflammatory cytokines (IL-1β and IL-6). It is well-known that DA exhibited an anti-inflammatory effect and is essential to control neuroinflammation (78). In this study, LP postbiotic pretreatment significantly suppressed the reduction of DA during ST infection, suggesting the pivotal role of DA in LP postbiotics-mediated anti-inflammation. In addition, we found a significant correlation between SCFAs and DA. SCFAs are important metabolites of the gut microbiota which possess various beneficial effects to the host (79). Accumulating studies have proved their ability in modulating brain functions as they can cross through the brain barrier and drive neurological functions (80). It was reported that SCFAs can activate DA biosynthesis and control its level by regulating tyrosine hydroxylase expression (81). In this study, LP postbiotics particularly LP metabolites markedly increased the concentrations of SCFAs (acetate, propionate), indicating that LP might modulate DA levels by increasing SCFAs. All the above findings demonstrated that LP postbiotics optimized the composition and SCFA production of the gut microbiota to inhibit neuroinflammation, which finally contributed to alleviating *Salmonella*-induced neurological dysfunction.

In summary, LP postbiotics (particularly the metabolites) ameliorated *Salmonella*-induced neurological dysfunctions by modulating gut–brain axis. This study highlights the effectiveness of postbiotics on improving neurological functions and provide a promising strategy for preventing infection-induced brain disorders by targeting gut microbiota.

## Data availability statement

The datasets presented in this study can be found in online repositories. The names of the repository/repositories and accession number(s) can be found below: https://www.ncbi.nlm.nih.gov/sra, PRJNA826309.

## Ethics statement

The animal study was approved by the Animal Care and Use Committee of Zhejiang Agricultural and Forestry University.

## Author contributions

CY supervised the project. YWu and CY designed the study. YWu drafted the manuscript. YWa, AH, XS, and WH conducted the experiments. YWu, AH, XS, and BW performed data analysis. CY, RZ, JL, and MY revised the manuscript. All authors have read and approved the final version of the manuscript.

## Funding

This study was supported by the National Natural Science Foundation of China (No. 32002212), Key R&D Program of Zhejiang Province (No. 2022C02043), Natural Science Foundation of Zhejiang Province (No. LQ21C170001), Zhejiang Provincial Key R&D Program of China (No. 2021C02008) and Zhejiang Provincial Leading Innovation and Entrepreneurship Team Project (No. 2020R01015).

## Conflict of interest

Author JL was employed by Zhejiang Vegamax Biotechnology Co., Ltd. The remaining authors declare that the research was conducted in the absence of any commercial or financial relationships that could be construed as a potential conflict of interest.

## Publisher's note

All claims expressed in this article are solely those of the authors and do not necessarily represent those of their affiliated organizations, or those of the publisher, the editors and the reviewers. Any product that may be evaluated in this article, or claim that may be made by its manufacturer, is not guaranteed or endorsed by the publisher.

## Abbreviations

LP: Lactobacillus. plantarum
ST: Salmonella. enterica Typhimurium
CNS: central nervous system
SCFAs: short-chain fatty acids
5-HT: 5-hydroxytryptamine
GABA: gamma-aminobutyric acid
BDNF: brain-derived neurotrophic factor
LDB: light–dark box test
OFT: open field test
RAM: Radial Arm Maze
FST: forced swim test
TST: tail suspension test
DA: dopamine
ACH: acetylcholine
NPY: neuropeptide Y
OTUs: operational taxonomic units
PCoA: principal co-ordinates analysis.
